# Differential Effects of Transition Metals on Growth and Metal Uptake for Two Distinct *Lactobacillus* Species

**DOI:** 10.1128/spectrum.01006-21

**Published:** 2022-01-26

**Authors:** Uyen Huynh, Muxin Qiao, John King, Brittany Trinh, Juventino Valdez, Marium Haq, Melissa L. Zastrow

**Affiliations:** a Department of Chemistry, University of Houston, Houston, Texas, United States; University of Maryland School of Pharmacy

**Keywords:** gut microbiota, *Lactobacillus*, *Lactobacillus plantarum*, *Lactobacillus acidophilus*, manganese, zinc, iron, metallobiology

## Abstract

*Lactobacillus* is a genus of Gram-positive bacteria and comprises a major part of the lactic acid bacteria group that converts sugars to lactic acid. *Lactobacillus* species found in the gut microbiota are considered beneficial to human health and commonly used in probiotic formulations, but their molecular functions remain poorly defined. Microbes require metal ions for growth and function and must acquire them from the surrounding environment. Therefore, lactobacilli need to compete with other gut microbes for these nutrients, although their metal requirements are not well-understood. Indeed, the abundance of lactobacilli in the microbiota is frequently affected by dietary intake of essential metals like zinc, manganese, and iron, but few studies have investigated the role of metals, especially zinc, in the physiology and metabolism of *Lactobacillus* species. Here, we investigated metal uptake by quantifying total cellular metal contents and compared how transition metals affect the growth of two distinct *Lactobacillus* species, Lactobacillus plantarum ATCC 14917 and Lactobacillus acidophilus ATCC 4356. When grown in rich or metal-limited medium, both species took up more manganese, zinc, and iron compared with other transition metals measured. Distinct zinc-, manganese- and iron-dependent patterns were observed in the growth kinetics for these species and while certain levels of each metal promoted the growth kinetics of both *Lactobacillus* species, the effects depend significantly on the culture medium and growth conditions.

**IMPORTANCE** The gastrointestinal tract contains trillions of microorganisms, which are central to human health. Lactobacilli are considered beneficial microbiota members and are often used in probiotics, but their molecular functions, and especially those which are metal-dependent, remain poorly defined. Abundance of lactobacilli in the microbiota is frequently affected by dietary intake of essential metals like manganese, zinc, and iron, but results are complex, sometimes contradictory, and poorly predictable. There is a significant need to understand how host diet and metabolism will affect the microbiota, given that changes in microbiota composition are linked with disease and infection. The significance of our research is in gaining insight to how metals distinctly affect individual *Lactobacillus* species, which could lead to novel therapeutics and improved medical treatment. Growth kinetics and quantification of metal contents highlights how distinct species can respond differently to varied metal availability and provide a foundation for future molecular and mechanistic studies.

## INTRODUCTION

The gut microbiota is a complex and dynamic microbial ecosystem, which is crucial for maintaining metabolic and immune homeostasis and protecting the host from pathogens (1–7). Different microbial species have distinct effects on these physiological functions, and changes in the microbial composition of the gut microbiota are often correlated with disease and infection (8, 9). Because hundreds of species in the gastrointestinal tract (GIT) are continuously competing for nutrients, changes in gut microbiota composition can be induced by factors like diet and drug treatment (10). *Lactobacillus* species are included in the lactic acid bacteria group, which comprises ∼0.01% to 1.8% of the total intestinal bacterial community (11, 12). These bacteria have complex nutritional requirements and form lactic acid as the sole or main product of carbohydrate metabolism (13, 14). Despite their relatively small numbers and that ∼25% of humans do not harbor stable populations of lactobacilli, this group of bacteria remains well-recognized for its importance to human health and utility in probiotic formulations (11, 15–18).

Among an array of nutrients, gut microbes must compete for metal ions, including transition metals like iron, zinc, and manganese, which can be acquired from the host diet (19). Transition metals are crucial to all living systems and function in processes ranging from catalysis to protein structure stabilization and cellular signaling (20). Lactobacilli are recognized for low iron requirements and high manganese uptake, but less is known about zinc requirements and function in these bacteria (21–26). The proportions of lactobacilli in the microbiota are often correlated with changes in dietary metal levels, sometimes varying between different species, but the underlying mechanisms are not understood (19, 27, 28).

Zinc, the second most abundant transition metal in biology, is required by all known organisms and has widespread roles, ranging from catalysis and structural stabilization to cellular signaling (29, 30). Few studies, however, have investigated the role of zinc in *Lactobacillus* species. Early work suggested toxic effects from adding excess zinc to growth medium and more recent work investigated the effects of zinc sulfate on a probiotic Lactobacillus plantarum strain and of zinc oxide (ZnO) in growth medium of several intestinal lactobacilli (31–34). Most lactobacilli exhibited high resistance to ZnO, but the underlying mechanisms were not explored (34). Several studies using animal models showed that zinc deficiency and supplementation affect the microbial composition of the gut microbiota and can be correlated with positive or negative impacts (35–49). Lactobacilli are often affected, but to varying degrees and sometimes with conflicting results (37, 38, 40–43, 45). The few studies that report on species-level changes show how different species can respond distinctly (37, 41). To the best of our knowledge, however, there are no reports investigating how varied zinc affects lactobacilli growth and zinc requirements for lactobacilli remain poorly defined. Microbiological and biochemical studies investigating the role of zinc in different *Lactobacillus* species will be required to understand their responses to varied zinc levels in the GIT.

Iron is the most abundant transition metal in biology, but early work on some species of lactobacilli showed that they could grow with little to no iron and did not acquire significant levels of iron (21–26). On the other hand, some species accumulated iron and others benefitted from heminic iron sources compared with commonly used iron salts (i.e., FeCl_3_, FeSO_4_) (50, 51). Although complex, several studies investigating how iron impacts the gut microbiota in humans, animals, and *in vitro*, have revealed iron-induced changes in lactobacilli (27, 52–63). One of the more consistent findings is an iron-induced decrease in lactobacilli and other beneficial bacteria along with an increase in *Enterobacteria* (a group including opportunistic species like Escherichia coli and Salmonella) (27, 52, 56). Furthermore, host iron status and iron levels can impact the gut microbiota composition and some lactobacilli can affect host iron sensing (64–66). Despite low iron requirements, lactobacilli are clearly affected by dietary iron levels and play roles in modulating host iron homeostasis. Low iron requirements could explain studies where iron-deficient conditions correlate with increased relative levels of lactobacilli (54, 57, 61–63), but given complex results from iron supplementation studies that could be due to differences in iron metabolism for different species and strains or to varied experimental and model conditions, more detailed studies on these bacteria and their interactions with iron are required.

In contrast to iron, lactobacilli take up high quantities of manganese compared with many bacteria (67–71). L. plantarum accumulates up to 20 mM manganese whereas bacteria like E. coli typically acquire manganese to μM levels (72). Manganese is crucial for managing oxidative stress, especially in lactobacilli grown under aerobic conditions, and may compensate for iron deprivation (68, 69, 73–75). Antioxidant effects conferred by non-proteinaceous manganese and manganese-bound superoxide dismutases and catalases are considered a possible beneficial effect of probiotic lactobacilli on the host (76–79). Few studies have directly investigated the role of manganese in the gut microbiota, but manganese supplementation in animals is linked with an increase in lactobacilli (80, 81). Another study, however, found no manganese-dependent changes in microbiome composition in a colitis mouse model (82). As for iron, there is a possibility for species and strain-dependent effects in response to varied manganese availability, and detailed studies are required.

Lactobacillus acidophilus and L. plantarum are among several species considered to permanently colonize the intestinal tract (83). Several studies reporting that lactobacilli have low iron and high manganese requirements focused on L. plantarum ATCC 14917 (21–25), but this work was prior to publication of many lactobacilli genome sequences, some of which reveal the presence of putative iron proteins, and many gut microbiota studies. L. acidophilus ATCC 4356 was previously found to accumulate Fe^2+^ ions and some strains acquire more zinc than several other *Lactobacillus* species (50, 84). One possible explanation is that the S-layer proteins found on the surface of L. acidophilus and not on L. plantarum may bind more metal ions and facilitate uptake (85–87). Given the above previous studies on L. acidophilus ATCC 4356 and L. plantarum ATCC 14917, and colonization of these species in the intestinal tract, we focused our studies on these two distinct strains of lactobacilli.

Here we set out to investigate how zinc, manganese, and iron affect growth of two distinct *Lactobacillus* species, L. plantarum and L. acidophilus, in controlled conditions with varied growth media. We report metal ion content for both species grown in rich and defined media, and show that among the transition metals studied, manganese was most abundant, then zinc and iron. We describe how growth kinetics of each species varies in nutrient-rich complex and metal-limited defined media and furthermore, how subculturing with fixed concentrations of metals or mucin affects the growth response to varied zinc, iron, or manganese in defined medium. The mucin glycoprotein is a primary component of the host mucus layer lining the GIT, and can bind metals (88, 89). Our results show that some subculture conditions produce distinct effects on the subsequent growth response to varied metal availability, supporting the idea that the local intestinal environment will influence how these bacteria survive within the gut microbiota. Additionally, excess zinc reduced the growth of L. acidophilus to a larger extent than L. plantarum. This work lays a foundation for detailed studies of the metallobiology of intestinal *Lactobacillus* species.

## RESULTS

### Quantitative metal uptake of *Lactobacillus* species and E. coli.

**FIG 1:**
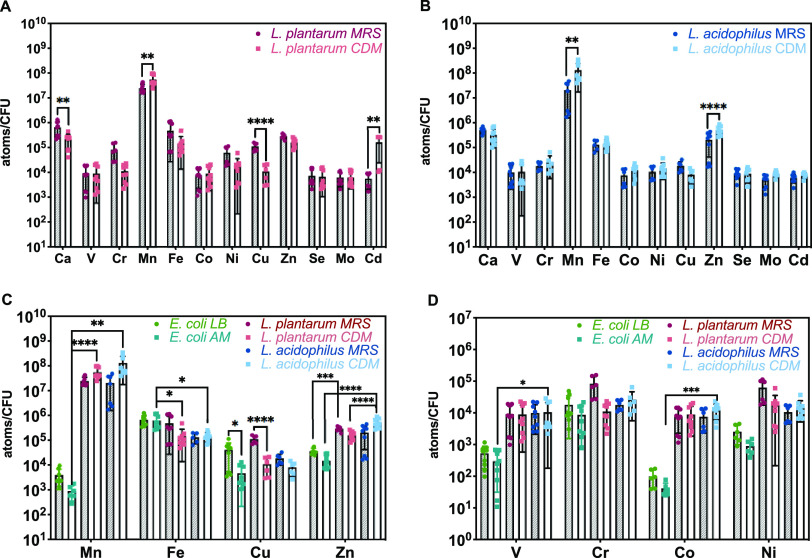
Metal contents of *Lactobacillus* species and E. coli in nutrient rich (MRS or LB) and metal-limited chemically defined minimal (AM or CDM) media as measured by ICP-MS. (A) *L. plantarum*, (B) L. acidophilus, and (C–D) comparison of essential metal uptake for E. coli and *Lactobacillus* species. Data are presented using a log-10 scale for the *y* axis. Error bars are SD of three biological replicates, each with ≥2 technical replicates. *P ≤ 0.05; **P ≤ 0.01; ***P ≤ 0.001; ****P ≤ 0.0001 as determined by one-way ANOVA with Tukey multiple comparison test.

We used rich (MRS) and chemically defined minimal (CDM) medium to investigate metal uptake of L. plantarum ATCC 14917 and L. acidophilus ATCC 4356 (90–92). MRS medium contains components that are not chemically defined and can have varied metal concentrations. We pretreated CDM medium components with Chelex to remove metals, and then trace metal grade Mn^2+^ was supplemented for aerobic growth of lactobacilli. This Chelexed CDM medium (Table S1, including the supplemented Mn^2+^) was used as the base minimal medium for all experiments using CDM medium except where Mn^2+^ was varied. Trace metal grade metals were added where indicated. Inductively coupled plasma-optical emission spectrometry (ICP-OES) analysis of MRS and CDM media confirmed low micromolar to subnanomolar levels of metal ions in CDM medium, including for iron and zinc (≤0.07 μM and ≤0.02 μM, respectively; Table S2). The metal concentrations in CDM allow for investigation of *Lactobacillus* growth and metal uptake under metal-limited conditions and are comparable with other Chelexed media (93, 94). To investigate metal uptake in these bacteria, we grew each strain in MRS overnight, subcultured in fresh MRS or CDM medium and harvested at mid-log phase. Cells were washed with mQ water and ethylenediaminetetraacetic acid (EDTA) to remove surface-bound metal ions, then dried, digested, and analyzed for intracellular metal contents using inductively coupled plasma-mass spectrometry (ICP-MS, Fig. 1, Table S3). We focused on essential elements but also investigated cadmium because previous studies revealed that Cd^2+^ and Mn^2+^ could compete for uptake through a P-type ATPase Mn^2+^ transporter (MntA) in L. plantarum (71, 95). We also quantified metal contents for E. coli K-12 BW25113 grown in rich (LB) and A-minimal (AM) media to compare with previous work and measured metal contents of lactobacilli (72). L. acidophilus and L. plantarum show minor differences in metal contents (Fig. 1A and B), and as previously reported, E. coli scavenges metal nutrients from metal-limited minimal medium (Fig. 1C and D, Fig. S1) (72). Both L. acidophilus and L. plantarum took up high amounts of manganese (∼2 × 10^7^ atoms/CFU) and more calcium, zinc, and iron (∼1 × 10^5^ to 7 × 10^5^ atoms/CFU) than other elements analyzed. L. acidophilus acquired slightly more zinc in CDM than MRS. Both lactobacilli acquired more manganese when grown in CDM compared with MRS, but this effect is more pronounced for L. acidophilus. ICP-MS analysis reveals significant iron content for both species. L. plantarum also took up more cadmium when grown in CDM than in MRS. For E. coli, iron contents are highest in both media, followed by substantial calcium and zinc quantities and E. coli accumulated significantly less manganese than L. plantarum and L. acidophilus (Fig. 1C, Fig. S1).

### Media-dependent effects on lactobacilli growth.

**FIG 2:**
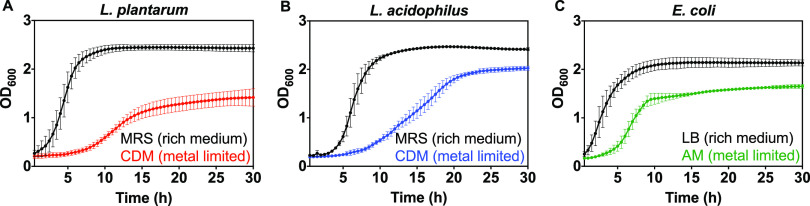
Growth kinetics of *Lactobacillus* species (A–B) and E. coli (C) in nutrient rich (MRS or LB, black circles) and metal-limited (CDM or AM) media and as measured by OD_600_. CDM growth curves are represented by red (L. plantarum) and blue (L. acidophilus) circles and AM represented by green circles. Error bars are SD of three biological replicates, each with ≥2 technical replicates.

**FIG 3:**
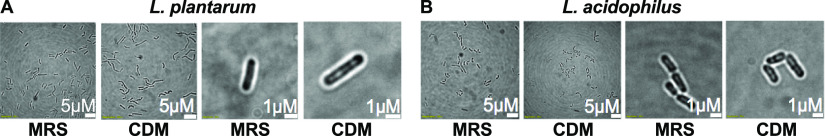
Bright-field microscopy images of (A) L. plantarum and (B) L. acidophilus when subcultured in MRS and CDM and harvested at stationary phase. Scale bars are 5 μm and 1 μm as labeled.

Given substantial uptake of manganese, zinc, and iron by L. plantarum and L. acidophilus grown in both media we explored the effects of these metals on growth kinetics. First, we investigated the effect of the medium on growth. Overnight *Lactobacillus* starter cultures (MRS) were washed and inoculated in MRS and CDM media. E. coli growth in LB and A minimal media were measured for comparison. All species grew more slowly and reached lower culture densities in metal-limited defined (CDM) compared with rich (MRS) medium. L. acidophilus showed a similar growth profile to L. plantarum in MRS, but showed more growth in CDM and reached a higher culture density in the stationary phase (Fig. 2). We also investigated cell morphology of lactobacilli in both media. Bright-field microscope images (Fig. 3) of the two species grown to stationary phase show no detectable differences in morphology or size.

### Zinc-dependent effects on growth of lactobacilli.

**FIG 4:**
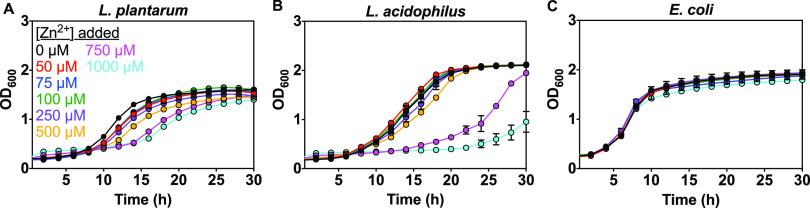
Effect of zinc on the growth of *Lactobacillus* species (A–B) in metal-limited medium (CDM) and E. coli (C) in minimal medium (AM). CDM and AM medium are each supplemented with 1% Wolfe trace mineral solution (see Materials and Methods). CDM is also supplemented with Mn^2+^. Representative growth curves are shown with error bars as SD from one biological replicate with ≥2 technical replicates.

**FIG 5:**
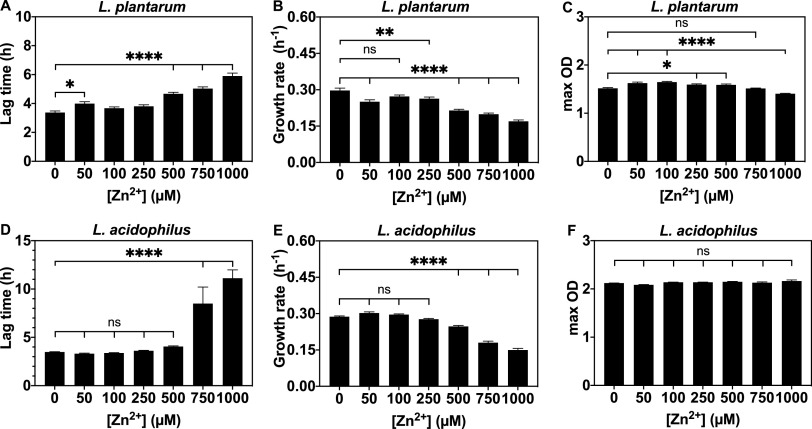
Zinc dependence of the growth parameters of *Lactobacillus* species grown in CDM (Table S1) supplemented with 1% Wolfe trace mineral solution containing all metals except zinc (see Materials and Methods). (A–C) L. plantarum and (D–F) L. acidophilus. Lag time, growth rate, and max OD represent the mean ± SEM of three biological replicates, each with ≥2 technical replicates. Growth parameters were calculated using nonlinear regression curve fitting to a four-parameter Logistic equation with GraphPad Prism 9 software. Ns, not significant; *P ≤ 0.05; **P ≤ 0.01; ****P ≤ 0.0001 as determined by one-way ANOVA with Tukey multiple comparison test.

Next, we investigated the effects of varied zinc on growth kinetics of each *Lactobacillus* species. We used MRS and supplemented the metal-limited CDM medium (Table S1) with other essential metals (Wolfe trace metal solution, Fig. S3) (96). Wolfe trace metal solution containing all metals except Zn^2+^ (final concentration 1%, see Materials and Methods) was supplemented to allow us to investigate the effects of varied Zn^2+^ only and avoid limiting multiple metals all at once. Overnight *Lactobacillus* starter cultures were washed and inoculated in fresh medium supplemented with varied zinc (0-1000 μM). Growth kinetics of E. coli in LB and AM media with varied zinc were also measured. Lactobacilli were not significantly affected by addition of zinc in MRS medium (Fig. S2), but Zn^2+^ significantly influenced growth in CDM medium (Fig. 4A and B). For E. coli, zinc showed minimal effects on growth kinetics regardless of the medium (Fig. 4C). Varied zinc primarily affected the lag time and growth rate for both *Lactobacillus* species, but not the maximum optical density at 600 nm (max OD) (Fig. 5). Higher added zinc concentrations (750 to 1,000 μM Zn^2+^) reduced the growth rate for both species (Fig. 5B and E) and more than doubled the lag time for L. acidophilus (Fig. 5D). To determine if the zinc source affects *Lactobacillus* growth, we compared zinc gluconate and zinc sulfate. Both salts showed generally similar effects on growth kinetics of L. plantarum (Fig. S4), so zinc sulfate was used for all other experiments.

**FIG 6:**
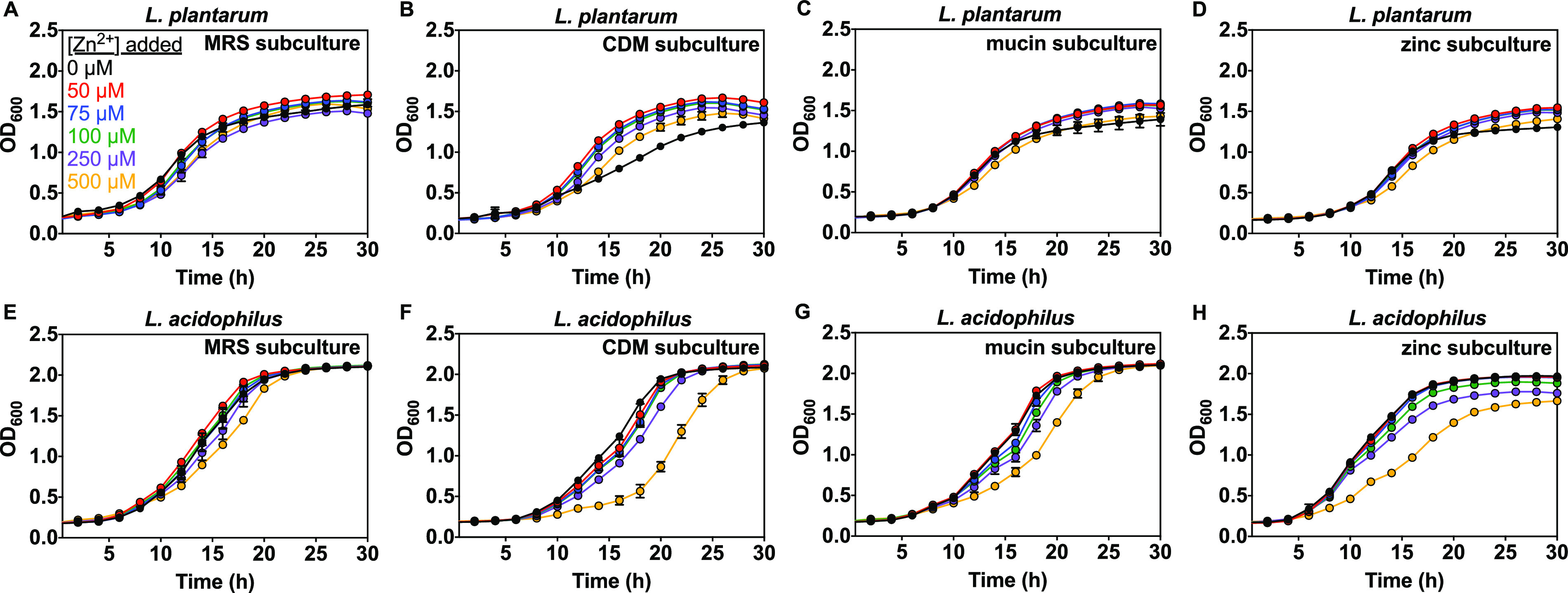
Effect of zinc on subculture growth for L. plantarum (A–D) and L. acidophilus (E–H) in CDM (Table S1) supplemented with 1% Wolfe trace mineral solution containing all metals except zinc (see Materials and Methods) and as measured by OD_600_. MRS subculture was grown in MRS. CDM subculture was grown in CDM. Mucin subculture was grown with 0.1% mucin in CDM. Zinc subculture was grown with 100 μM ZnSO_4_ in CDM (Scheme S1). Each of the above subcultures was then washed and grown in CDM (Table S1) supplemented with 1% Wolfe trace mineral solution containing all metals except zinc. Representative growth curves are shown with error bars as SD from one representative biological replicate with ≥2 technical replicates.

Given that zinc affects the growth kinetics of both *Lactobacillus* species in CDM medium, we explored the effects of zinc on lactobacilli subcultured under various conditions. We hypothesized that varied subculture conditions could result in different basal proteomes, and lead to differences in how zinc affects growth. For example, bacteria grown in nutrient- and metal-limited medium (CDM) could have different protein abundance profiles compared to the same bacteria grown in MRS or CDM supplemented with mucin or zinc. Mucin, a glycoprotein, is a primary component of the protective mucus layer in the GIT and can bind metals (88, 89). Lactobacilli may adhere to mucin using various surface proteins (97, 98). We hypothesized that including mucin in growth medium could affect expression of surface proteins and possibly influence the response to growth with varied metal concentrations. Here we systematically grew lactobacilli starter cultures in MRS overnight then washed and subcultured each species overnight in MRS, CDM only, or CDM medium supplemented with 0.1% mucin or 100 μM ZnSO_4_. After overnight incubation, subcultures were washed and inoculated in fresh medium supplemented with varied zinc (0-500 μM Zn^2+^) and incubated again for growth kinetics monitoring (Scheme S1). For each species subcultured in MRS, we monitored growth kinetics in CDM supplemented with 1% Wolfe trace mineral solution and found that the effects of zinc were similar (Fig. 6, Fig. S5, S6) to those for samples that were not subcultured (Fig. 4 and 5). Therefore, the additional subculture step in MRS does not change the response to zinc or negatively influence growth. Conversely, when each species was subcultured in CDM, mucin, or zinc, and then grown in CDM supplemented with Wolfe solution, growth kinetics were significantly affected by zinc levels. Note that for subculture studies, we narrowed down the zinc range to 0 to 500 μM Zn^2+^ to exclude the more toxic 750 and 1,000 μM concentrations used above. The luminal zinc concentration in the small intestine (where most zinc absorption occurs) is estimated to be 10 to 250 μM, and although local variations are likely much lower during infection, the 0 to 500 μM range should cover physiologically relevant levels (99–101). When L. plantarum was subcultured in CDM, the growth kinetics as a function of zinc shows shortened lag time and increased growth rate at 50 to 250 μM compared with no added Zn^2+^, and decreased growth rate at 500 μM (Fig. 6B, Fig. S5B). For L. plantarum subcultured in the presence of mucin or 100 μM Zn^2+^; however, 50 to 250 μM Zn^2+^ had no significant impact on lag time, growth rate, and max OD, (Fig. 6C and D, Fig. S5C, S5D, Table S4). When L. acidophilus was subcultured in CDM no significant impact on lag time, growth rate, and max OD was observed at 50 to 100 μM Zn^2+^ but at 500 μM zinc an increased lag time, from 3.8 h to 4.8 h, and reduced growth rate, from 0.26 to 0.21 h^-1^, were measured and from 250 to 500 μM zinc, a lower max OD was observed (Fig. 6F, Fig. S6B). In the presence of mucin or zinc in the subculture growth medium (CDM), the same trend was observed, but there was a more significant delay in lag time and reduction in the growth rate at 500 μM zinc by comparison to the CDM subculture (Fig. 6G and H, Fig. S6C, S6D, Table S5). Analogous subculture conditions were used to investigate E. coli and revealed that zinc did not affect growth of LB, AM, mucin (0.1%), or zinc (100 μM) subcultures (not shown).

### Manganese-dependent effects on growth of lactobacilli.

**FIG 7:**
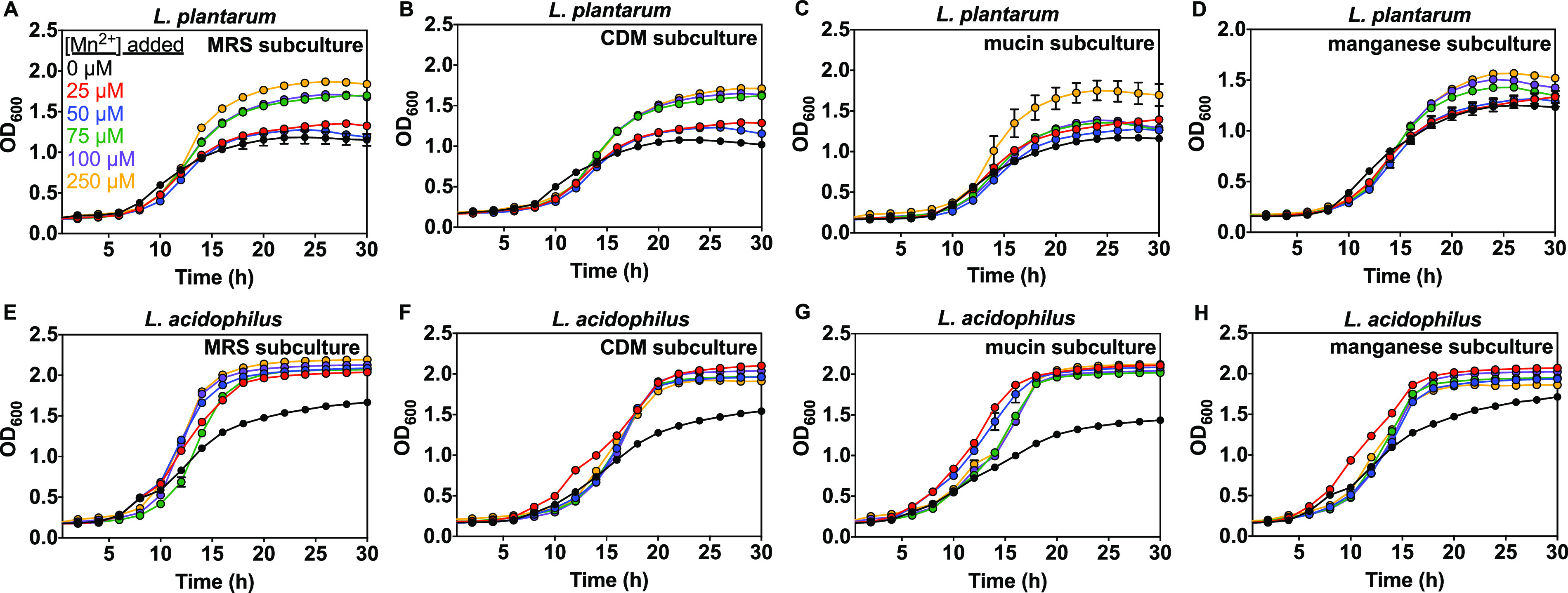
Effect of manganese on subculture growth for L. plantarum (A–D) and L. acidophilus (E–H) in CDM prepared without added manganese (Table S1) supplemented with 1% Wolfe trace mineral solution containing all metals except manganese (see Materials and Methods) and as measured by OD_600_. MRS subculture was grown in MRS. CDM subculture was grown in CDM. Mucin subculture was grown with 0.1% mucin in CDM. Manganese subculture was grown with 50 μM MnCl_2_ in CDM (Scheme S1). Each of the above subcultures was then washed and grown in CDM (Table S1) supplemented with 1% Wolfe trace mineral solution containing all metals except manganese. Representative growth curves are shown with error bars as SD from one representative biological replicate with ≥2 technical replicates.

Given the high manganese requirements of lactobacilli, we investigated the effect of manganese on growth kinetics of L. plantarum and L. acidophilus. Concentrations were varied around an estimated physiological range for the small intestine (0 to 250 μM) (102, 103). Here we used trace mineral supplemented CDM medium containing all metals outlined in the Wolfe solution except Mn^2+^ and including Zn^2+^. Overnight starter cultures of *Lactobacillus* (MRS) were washed then subcultured in MRS, CDM, or CDM with 0.1% mucin or 50 μM MnCl_2_ (Scheme S1). Growth kinetics of the subcultures were measured in CDM with 0 to 250 μM Mn^2+^. As observed in previous studies (68–70), manganese promotes growth, but our results show that the sensitivity toward manganese varies between species and subcultures (Fig. 7, Fig. S7 and S8, Table S4 and Table S5). Growth of *Lactobacillus* species (MRS subcultures) in MRS medium was not affected by manganese (not shown) but 25 to 250 μM Mn^2+^ promoted growth in supplemented CDM (Fig. 7). Manganese also promoted growth for CDM, mucin, and manganese subcultures of both species, but added Mn^2+^ promoted L. acidophilus growth more strongly based primarily on the max OD (Fig. 7, Fig. S7 and S8, Table S4 and S5). For L. plantarum, 25 to 250 μM Mn^2+^ does not have a significant impact on the lag time and growth rate for most subcultures, with the exception of the mucin subculture grown in the presence of 250 μM added Mn^2+^, which had a shorter lag time (3.1 h versus 4.3 h for 0 μM Mn^2+^) and increased growth rate (0.32 h^−1^ versus 0.23 h^−1^). For L. acidophilus, however, CDM, mucin, and Mn^2+^ subcultures had a shorter lag time and faster growth rate, especially at 100 to 250 μM Mn^2+^, compared with MRS subcultures (Fig. 7, Fig. S8). Most L. plantarum subcultures only showed an increase in max OD at 100 to 250 μM Mn^2+^ (Fig. S7) whereas all L. acidophilus subcultures achieved significantly higher max ODs when grown in the presence of 25 to 250 μM Mn^2+^ compared with no added Mn^2+^ (Fig. S8).

### Iron-dependent effects on growth of lactobacilli.

**FIG 8:**
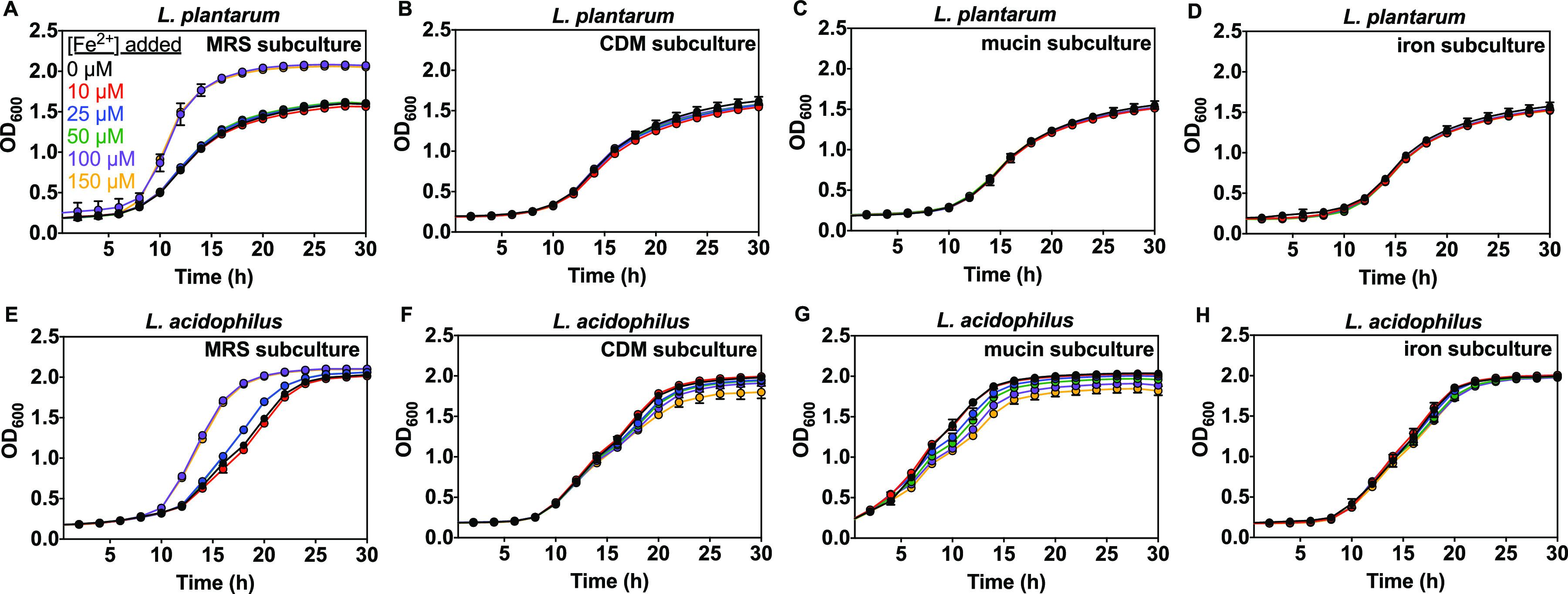
Effect of iron on subculture growth for L. plantarum (A–D) and L. acidophilus (E–H) in CDM (Table S1) supplemented with 1% Wolfe trace mineral solution containing all metals except iron (see Materials and Methods) and as measured by OD_600_. MRS subculture was grown in MRS. CDM subculture was grown in CDM. Mucin subculture was grown with 0.1% mucin in CDM. Fe subculture was grown with 25 μM FeSO_4_ in CDM (Scheme S1). Each of the above subcultures was then washed and grown in CDM (Table S1) supplemented with 1% Wolfe trace mineral solution containing all metals except iron. Representative growth curves are shown with error bars as SD from one representative biological replicate with ≥2 technical replicates.

We investigated the effect of iron varied from 0 to 150 μM based on an estimated jejunal physiological range (104, 105). We used a similar method as for zinc and manganese, but with Wolfe solution containing 100 μM Zn^2+^ and no Fe^2+^. Overnight starter cultures were washed and subcultured in MRS, CDM, CDM supplemented with 0.1% mucin or 25 μM FeSO_4_ then inoculated into growth medium with varied iron (0 to 150 μM Fe^2+^, Scheme S1). Growth of both *Lactobacillus* species from MRS subcultures was mostly not affected by iron, except 100 to 150 μM added iron boosted the growth rate from 0.23 to 0.35 h^−1^ (L. plantarum) and from 0.25 to 0.36 h^−1^ (L. acidophilus), shortened the lag time from 4.4 to 2.9 h (L. plantarum) and from 4.0 to 2.8 h (L. acidophilus), and resulted in higher max OD (1.78 compared with 1.6) for L. plantarum (Fig. 8 and Fig. S9, S10). On the other hand, growth of CDM, mucin, and iron subcultures was insensitive to the iron present in the medium, except for the mucin subculture of L. acidophilus, which exhibited a slight delay in the lag time and suppression of the growth rate and max OD with increasing iron (Fig. 8, Fig. S9, S10, Table S4 and S5).

### Growth kinetics of *Lactobacillus* species in zinc-, manganese-, and iron-deprived conditions.

**FIG 9:**
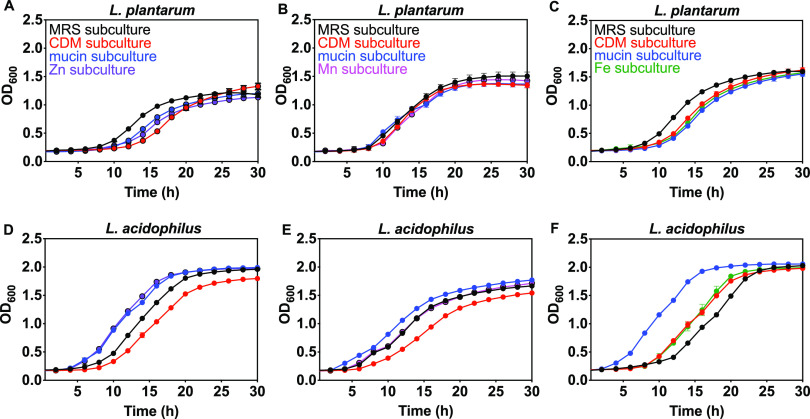
Effect of zinc-, manganese-, and iron-deprivation on subculture growth for L. plantarum (A–C) and L. acidophilus (D–F) in CDM supplemented with 1% Wolfe solution as measured by OD_600_. CDM (Table S1 or Methods) used for zinc deprivation is supplemented with 1% Wolfe trace mineral solution containing all metals except zinc. CDM used for manganese deprivation is prepared without added manganese and supplemented with 1% Wolfe trace mineral solution containing all metals except manganese. CDM used for iron deprivation is supplemented with 1% Wolfe trace mineral solution containing all metals except iron. MRS subculture was grown in MRS. CDM subculture was grown in CDM. Mucin subculture was grown with 0.1% mucin in CDM. Zn subculture was grown with 100 μM ZnSO_4_ in CDM. Mn subculture was grown with 50 μM MnCl_2_ in CDM. Fe subculture was grown with 25 μM FeSO_4_ in CDM (Scheme S1). Representative growth curves are shown with error bars as SD from one representative biological replicate with ≥2 technical replicates.

We also compared how lactobacilli subcultures respond to zinc-, manganese-, or iron-deprived conditions. Subculture growth kinetic data was collected and described above, and here we compare growth curves representing the no added metal conditions when subcultures were inoculated in supplemented CDM (Fig. 9). The zinc concentration in the zinc-limited condition should therefore be no greater than ∼0.02 μM as quantified using ICP-OES, the concentration of manganese should be <0.01 μM (this value is based on measured water since CDM medium in Table S2 contains added MnCl_2_), and iron should be 0.07 μM. The mineral supplement for the manganese-deprived condition contains 100 μM Zn^2+^ but no Mn^2+^ and the supplement for the iron-deprived condition contains no iron and 100 μM Zn^2+^. The CDM subculture of L. plantarum has a significantly delayed lag time and reduced growth rate compared with all other subcultures under zinc-deprived conditions (Fig. 9 and Table S4). A similar result was obtained for L. acidophilus (Fig. 9 and Table S5). The zinc subculture of L. acidophilus, however, showed the shortest lag time and highest growth rate. For manganese deprivation, the shortest lag times and highest growth rates were observed for the MRS subculture (L. acidophilus) and for iron deprivation, the highest growth rate corresponds to the mucin subculture (L. acidophilus).

### Effect of media and metals on size and morphology of lactobacilli.

**FIG 10:**
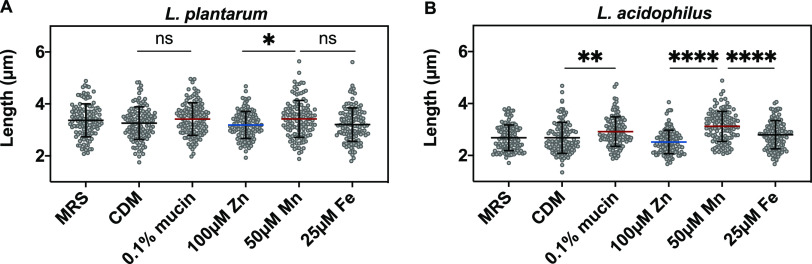
Length of (A) L. plantarum and (B) L. acidophilus cells grown in rich medium (MRS), metal-limited medium (CDM), and CDM supplemented with 0.1% mucin, 100 μM ZnSO_4_, 50 μM MnCl_2_, or 25 μM FeSO_4_. Error bars are SD of two biological replicates with a total of ∼120 cells for each condition. Ns, not significant; *P ≤ 0.05; **P ≤ 0.01; ****P ≤ 0.0001 as determined by one-way ANOVA with Tukey multiple comparison test.

Given the preferential uptake of manganese, zinc, and iron, and the effects of these metals on *Lactobacillus* growth kinetics, we examined whether media composition and mucin and metal supplements affect the morphology of L. plantarum and L. acidophilus. We grew and subcultured each *Lactobacillus* species as in the growth kinetics studies. Microscope samples were collected at the stationary phase (22 h to 24 h) after overnight subculturing and observed using bright-field microscopy. Images of these samples show that the size (Fig. 10) and morphology of lactobacilli were not significantly affected by media composition or presence of supplemented metals or mucin (Fig. S11).

## DISCUSSION

Dietary metals alter the composition of the gut microbiota and are often correlated with changes in relative abundance of *Lactobacillus* species, but underlying mechanisms and effects on individual species remain unclear (19, 27, 28). Metal homeostasis mechanisms are well understood for model organisms such as E. coli and B. subtilis as well as many pathogens, but lactobacilli have received comparatively little attention (101, 106, 107). This work provides insight to how iron, zinc, and manganese differentially affect the growth kinetics of two *Lactobacillus* species under systematically varied culture conditions. Early work explored roles and requirements for manganese and iron in lactobacilli, but this was primarily before genomic data was available and studies focused on zinc are severely lacking (21–25). ICP-MS quantification of cellular metal content revealed that L. plantarum and L. acidophilus acquired more manganese than any other metal measured, followed by calcium, zinc, and iron, and that E. coli takes up significantly less manganese (Fig. 1, Table S3). These results are consistent with previous work establishing that *Lactobacillus* species like L. plantarum require millimolar levels of manganese for protection against oxygen toxicity (68, 69, 74, 75). L. plantarum and L. acidophilus acquired slightly more manganese in CDM medium, suggesting increased manganese requirements in nutrient-limited conditions. Zinc contents of L. plantarum were similar in both media, but L. acidophilus accumulated more zinc when grown in CDM, suggesting a higher zinc requirement in nutrient-limited conditions, too. Previous work showed that among several *Lactobacillus* species, two strains of L. acidophilus including ATCC 4356 accumulated the highest cell-bound zinc concentrations (84). Although this study focused on surface-bound zinc, our data suggest these results could apply to overall zinc content for this species. We also detected substantial iron levels in L. plantarum and L. acidophilus in both media. Previous work recorded either very little iron uptake in the same strain of L. plantarum or some Fe^2+^ accumulation in both species, but the growth conditions were different than those in this current study (21, 24, 50). Specifically, this work varies from previous studies in composition of growth media, the growth phase at which samples were collected, whether bacteria were incubated in anaerobic or aerobic conditions, which could lead to different CO_2_ metabolism (108, 109), and in carbon source or presence of glucose (110–112).

Both species acquired cadmium from MRS and CDM media. L. acidophilus shows similar uptake in both media, but cadmium contents of L. plantarum are significantly increased in CDM, as was also observed for manganese, and is consistent with literature on L. plantarum uptake transporters (71, 95). Cadmium uptake was first detected in L. plantarum ATCC 14917, where Cd^2+^ was preferentially taken up by a Mn^2+^ transporter and both Mn^2+^ and Cd^2+^ uptake were significantly induced by Mn^2+^-starvation (71). MntA, the transporter responsible, was later identified and while Cd^2+^ and Mn^2+^ competitively inhibit each other, MntA appears to have higher affinity for Cd^2+^ (95). No manganese or cadmium transporters have been experimentally identified for L. acidophilus.

Given that manganese, zinc, and iron can all affect lactobacilli abundance in various gut microbiota studies (27, 28) and that both L. plantarum and L. acidophilus accumulate significant quantities of these metals, the effects on *Lactobacillus* growth kinetics were examined and compared to E. coli. Despite evidence that dietary zinc abundance affects *Lactobacillus* levels in the gut microbiota, there are few studies investigating how zinc affects *Lactobacillus* growth kinetics (27, 28, 34). Varied zinc affects *Lactobacillus* growth in minimal but not rich medium (Fig. 4) but Zn^2+^ did not affect E. coli in either type of medium. L. acidophilus is more sensitive to zinc in the defined media than L. plantarum given that 750 to 1,000 μM Zn^2+^ increases L. acidophilus lag time more so than for L. plantarum (Fig. 4 and 5). This result could be explained by the fact that L. acidophilus acquires more zinc than other lactobacilli and has surface S-layer proteins, which might bind zinc and lead to higher accumulation than for L. plantarum and, therefore, more severe zinc-induced growth suppression (84–87). Including a trace mineral supplement in CDM medium can promote the growth rate of L. plantarum (at 0 μM Zn^2+^) and L. acidophilus (at 0 to 500 μM Zn^2+^) and significantly reduce the lag time of L. acidophilus at high Zn^2+^ (Fig. S3). One explanation for this result is that supplemented CDM provides additional essential metals besides zinc that may compete for uptake and reduce excessive accumulation of zinc ions.

The conditions experienced by bacteria in the gut microbiota could vary from those in pure culture due to varied nutrient availability and competition with other microbes. To explore how the growth environment affects zinc-dependent growth kinetics for *Lactobacillus*, each species was subcultured in rich or defined minimal growth media, and in the presence of mucin or supplemented metal. These varied growth conditions could lead to different proteomic profiles for bacteria, which may alter the impact of metals on growth kinetics if differentially expressed proteins are involved in metal-dependent cellular activities (e.g., metal uptake, transport, storage, metabolism, catalysis). For example, when E. coli is grown in medium with restricted iron availability, an increase in relative abundance of iron-associated transport proteins was detected (113). Proteomic profiles of several L. plantarum strains vary between growth media and affect carbohydrate utilization and energy metabolism, including a probable manganese-dependent inorganic pyrophosphatase (114). MRS subcultures of each *Lactobacillus* species subsequently grown in CDM showed no significant difference in zinc-dependent growth kinetics compared with the original culture grown in CDM (Fig. 6A and E, Fig. S5A, S6A) and there is no zinc effect on growth of lactobacilli in MRS (not shown). The zinc-dependent growth kinetics vary, however, for CDM, mucin, and zinc subcultures and between species (Fig. 6, Fig. S5, S6). L. plantarum subcultured in the presence of mucin or 100 μM Zn^2+^ had no significant impact on lag time, growth rate, and max OD at 50 to 250 μM Zn^2+^, but in CDM subcultures shortened lag times and increased growth rates were observed at the same range of zinc concentrations (Fig. 6B to D, Fig. S5B–5D). On the other hand, growth of all subcultures of L. acidophilus were suppressed significantly at 500 μM Zn^2+^, as evidenced by longer lag times and slower growth rates (Fig. 6E to H, Fig. S6A–6D, Table S4, S5). Growth of CDM, mucin, and zinc were also somewhat affected at 250 μM Zn^2+^, whereas the MRS subculture was not. These effects of zinc observed on the growth of L. acidophilus subcultures and the fact that L. acidophilus accumulates more zinc when grown in CDM than MRS (Fig. 1B) suggests increased expression or activity of proteins involved in zinc uptake for CDM, mucin, and Zn^2+^ subcultures. Increased zinc accumulation might lead more readily to toxic zinc overload and suppression of growth. Increased zinc accumulation could also be the result of reduced zinc export. The genome of L. plantarum contains annotated genes for the zinc export systems CadA and CzcD, and L. acidophilus contains only one gene annotated for CadA (115–117). Although not experimentally characterized in lactobacilli, these genes play important roles in conferring zinc resistance to the Gram negative organism, Pseudomonas aeruginosa (118, 119). In contrast to lactobacilli, zinc does not affect E. coli growth in rich or minimal media from starter cultures (Fig. 4C) or subcultures (minimal medium with or without mucin or zinc, not shown). Under zinc-deprived conditions, CDM subcultures of L. plantarum showed the longest lag time and slowest growth rate compared with other subcultures, which generally had similar parameters (Fig. 9A and Table S4). Zinc subcultures of L. acidophilus, however, grew with shorter lag times and higher growth rates than MRS, mucin, and CDM subcultures (Fig. 9D and Table S5). This result suggests that zinc conditioning from the subculture step could facilitate improved survival of L. acidophilus when transferred to zinc-deficient conditions. Further investigation is needed to understand how subculturing cells in zinc helps L. acidophilus cells to better tolerate zinc-deficient conditions.

Given the high manganese requirements of lactobacilli, MRS, CDM, and mucin and manganese subcultures were used to investigate Mn^2+^-dependent growth kinetics. As for zinc, manganese only affected growth in defined medium with no added Mn^2+^ (Fig. 7, Fig. S7, S8), and not in rich medium already containing high Mn^2+^ (not shown). L. plantarum and L. acidophilus growth were both promoted by 25 to 250 μM Mn^2+^, and both species achieved significantly higher biomass at stationary phase when grown in Mn^2+^-supplemented subculture medium compared with manganese-deprived medium, supporting the importance of manganese for lactobacilli (Fig. 7). The positive effect of 250 μM Mn^2+^ in defined medium is most pronounced for L. plantarum mucin subcultures as evidenced by a significantly shorter lag time, faster growth rate, and higher max OD compared to those lower Mn^2+^ concentrations (Fig. S7C and Table S4), suggesting a relationship between mucin and manganese uptake in L. plantarum. When grown in manganese-deprived conditions, there is no difference in the growth kinetics between all four subcultures of L. plantarum (Fig. 9), but the mucin subcultures of L. acidophilus consistently achieved slightly shorter lag times than other subcultures within each biological replicate. Subculturing L. acidophilus in mucin appears to help this species better adapt to manganese-deprived medium. Further studies, such as proteomic analysis, could reveal mechanistic details underlying how different growth conditions help some species better tolerate manganese deprivation. Because manganese is crucial for lactobacilli defense against oxygen toxicity (68, 69, 74, 75), it is important to investigate whether similar effects are observed under anaerobic conditions. Previous studies have shown decreased manganese reduces lactobacilli growth and some species undergo morphology and surface protein changes with manganese deprivation, along with global proteomic changes (120, 121).

We also studied how iron affects growth of both lactobacilli using analogous MRS, CDM, and mucin and iron subcultures (Fig. 8, Fig. S9, S10). Surprisingly, we found that Fe^2+^ (100 to 150 μM) promotes growth of L. plantarum and L. acidophilus MRS subcultures in supplemented CDM medium (Fig. 8A and E), but not in MRS (not shown). All other subcultures showed no iron effect on growth (Fig. 8, Fig. S9B–9D and S10B–10D). These results suggest that subculturing either species in MRS may affect expression of iron-associated proteins that alter iron uptake or metabolism when subsequently cultured in metal-limited medium. Results from previous studies reporting the lack of iron requirement in *Lactobacillus* species are in agreement with this study, except that these results reveal growth promotion (shorter lag time and higher growth rate) for MRS subcultures at higher iron concentrations than previously studied (100 to 150 μM) (21–23, 25, 26). Here there was no effect of iron on *Lactobacillus* growth at concentrations below 100 μM (Fig. S9, S10), and the effect was only observed in MRS subcultures grown in metal-limited medium. Previous work showed that iron can affect Lactobacillus johnsonii growth depending on the nucleotide sources used in defined medium (23). Another species, Lactobacillus sakei 23K, uses iron from heminic sources to lengthen survival in the stationary phase (51). The genome of this species and others contain genes likely involved in iron or heme transport (122–124). Taken together, our results are consistent with the existing literature that lactobacilli have a minimal requirement for iron but can accumulate it.

We also compared metal-limitation effects on the growth response of different lactobacilli subcultures and found that the mucin subculture of L. acidophilus showed more robust growth (shorter lag time and faster growth rate) than other subcultures and improved survival upon exposure to iron-limitation (Fig. 9 and Table S5). This result could indicate an association between mucin and surface proteins regulating metal uptake and binding for L. acidophilus. L. acidophilus NCFM (a strain 99.96% similar to ATCC 4356) grown in the presence of mucin showed increased abundance of surface proteins such as pyruvate kinase (PK) and fructose-bisphosphate aldolase (FBA) (116, 117, 125). PK is a key enzyme in glycolysis, and was upregulated for E. coli grown in iron-limiting conditions (126). FBA might contribute to intestinal cell adhesion in L. acidophilus L-92 (127).

The present work offers insight to how zinc, manganese, and iron affect the growth of two distinct *Lactobacillus* species under varied conditions in complex and defined media. Metal contents of L. plantarum and L. acidophilus grown in rich and minimal media were mostly similar but revealed increased uptake for some metals in nutrient-limited medium. This work uncovers distinct patterns for how zinc, manganese, and iron affect the growth kinetics of lactobacilli while E. coli is not affected under similar growth conditions. These observations support future studies into the molecular mechanisms underlying metal-dependent growth kinetics and metal uptake in lactobacilli and will contribute to understanding how essential metals affect intestinal lactobacilli and related probiotic organisms in the gut microbiota.

## MATERIALS AND METHODS

### General considerations.

All reagents were purchased from commercial sources and used as received. Trace metals and amino acids are BioXtra (>99.9%) and BioUltra (>99.5%) grade from Sigma-Aldrich. Aqueous solutions were prepared using Milli-Q water. To remove metal ions, solutions were treated with Chelex-100 resin (Bio-Rad) according to the manufacturer’s batch protocol. Chelex-treated solutions were stored in acid-washed plastic containers and transferred using acid-washed pipet tips. Growth curve experiments were monitored using a multimode microplate reader (Tecan Spark 10M) with 96-well clear round-bottom plates (Greiner Bio-One).

### Bacterial strains and culture conditions.

The bacterial strains used in this study are L. plantarum ATCC 14917, L. acidophilus ATCC 4356, and E. coli K-12 BW25113 (Keio knockout collection, Dharmacon, Inc.). E. coli was routinely cultured aerobically in Luria-Bertani (LB) medium at 37°C, and *Lactobacillus* species aerobically with 5% CO_2_ in De Man, Rogosa, and Sharpe (MRS) medium (90) at 37°C, both with shaking. Lactobacilli strain stocks were stored in MRS broth containing 25% (vol/vol) glycerol at −80°C and E. coli stocks in LB broth with 25% (vol/vol) glycerol at −80°C. Minimal media were used to study metal effects, specifically A minimal (AM) medium for E. coli BW25113 and CDM medium for *Lactobacillus* species (recipes below).

### Media and supplements.

LB broth powder (10 g/L tryptone, 5g/L yeast extract, 10g/L sodium chloride) for E. coli was purchased from Fisher Scientific. LB/agar plates were prepared using LB broth powder (25 g/L) and bacteriological agar (20 g/L, Sigma-Aldrich). AM medium was prepared according to published protocols and treated with Chelex (128). Chelex-treated AM medium was supplemented with Chelex-treated 0.2% glucose and 26.2 mM all 20 natural L-amino acids in equimolar quantities, each at 1.33 mM. MRS broth powder for *Lactobacillus* species was purchased from Sigma-Aldrich and contains 2 g/L dipotassium hydrogen phosphate, 20 g/L glucose, 0.2 g/L magnesium sulfate heptahydrate, 0.05 g/L manganous sulfate tetrahydrate, 8 g/L meat extract, 10 g/L peptone, 5 g/L sodium acetate trihydrate, 2 g/L triammonium citrate, 4 g/L yeast extract. MRS broth were prepared using MRS powder (51 g/L, Sigma-Aldrich) and 1 mL polysorbate 80 (Tween 80, Sigma-Aldrich). MRS/agar plates were prepared using MRS agar powder (61 g/L, Sigma-Aldrich) and 1 mL polysorbate 80 (Tween 80, Sigma-Aldrich). CDM medium was adapted from McFeeters et al. (91), using the amino acid concentrations from Wegkamp et al. (92), and was prepared from several Chelex-treated or trace metal reagent-containing stock solutions (Table S1). Wolfe solution (96) was used as a trace mineral supplement for cultures grown in CDM medium, and contains 1.5 g/L NTA, 1 g/L NaCl, 0.1 g/L FeSO_4_, 0.1 g/L Co(NO_3_)_2_, 0.1 g/L CaCl_2_, 0.01 g/L CuSO_4_, 0.01 g/L H_3_BO_3_, 0.01 g/L Na_2_MoO_4_, 0.001 g/L Na_2_SeO_3_, 0.01 g/L Na_2_WO_4_, 0.02 g/L NiCl_2_. This Wolfe trace mineral solution is used for zinc studies. The Wolfe solution used for manganese studies contains 0.02 g/L ZnSO_4_ and all metals described above. The Wolfe solution used for iron studies contains 0.02 g/L ZnSO_4_ and all metals described earlier except iron. Trace metal grade salts were used for components that could not be treated with Chelex. FeSO_4_ solutions were prepared fresh on each day of use.

### ICP-OES for metal content in growth medium.

Media samples (1.0 mL) were boiled in 2.0 mL metal-free plastic centrifuge tubes (Eppendorf). Dried sample from 1 mL of media (MRS or CDM) was digested with 280 μL Milli-Q water and 15 μL trace metal concentrated nitric acid at 80°C for at least 12 h. Digested samples from a total of 8 mL media were diluted with 2% trace metal HNO_3_ to bring to a total volume of 3 mL. Sample was stored at 4°C until analysis by ICP-OES.

Medium samples were analyzed by an Agilent Simultaneous 725 ICP-OES equipped with VistaChip II CCD detector and image mapping technology (I-MAP) to provide complete wavelength coverage from 167 to 785 nm. Trace metal HNO_3_ (2%) was used as blank. Metal concentrations in media were measured by ICP-OES for Ca, V, Cr, Mn, Fe, Co, Ni, Cu, Zn, Se, Mo, and Cd. ICP-OES calibration standards contain 5,000, 2,000, 1,000, 750, 500, 250, 100, 40, 25, 16, 10, 5, 2.5, 1, and 0.5 ppb for each metal and were all prepared by diluting commercially available Inorganic Ventures' ICP-MS Complete Standard 71A (10 ppm) and Inorganic Ventures' ICP-MS Refractory Elements Standard 71B (10 ppm) with 2% trace metal HNO_3_.

### ICP-MS for metal content in bacterial cells.

To determine intracellular metal levels, each *Lactobacillus* species was grown overnight with shaking (20 h) in MRS medium (10 mL) from an MRS/agar streak plate. Overnight cultures were diluted 1:100 into fresh MRS medium (150 mL) or washed then diluted into fresh CDM medium (150 mL) in a 250-mL acid-washed and autoclaved polycarbonate baffle flask. This experiment was performed in triplicate. Control flasks with 150 mL MRS or CDM but no added bacteria were prepared in parallel and subjected to all the same treatments as bacterial samples. Cells were grown with shaking to OD_600_ ∼0.6 to 0.9 (mid-log phase). Prior to harvesting cells for analysis, 50 μL of each sample was collected to perform serial dilution in 0.85% saline solution (in triplicate) for determining the CFU count of each cell sample. For each sample, serial dilution from 10^−1^ to 10^−5^ was prepared by adding 50 μL of each original solution into 450 μL of 0.85% saline solution in 1.5 mL-centrifuge tubes. Then, 15 μL of each dilution was plated on MRS agar plate (in triplicate) and incubated overnight. CFU/mL were determined through manual counting and by using ImageJ software (129). Harvested cell pellets were washed with fresh media, followed by washes using Milli-Q water (1x), 1 mM EDTA (3x), and Milli-Q water (1x). Pellets were dried overnight at 80°C in Teflon tubes, weighed, and digested in 100 μL trace metal grade concentrated nitric acid at 65°C for 30 min, then increased to 100°C for 5 h. Digested cells and control samples were subsequently diluted with 1.4 mL Milli-Q water and transferred to a 15 mL-metal-free centrifuge tube. Each Teflon tube was then washed with 1 mL of Milli-Q water which was then transferred to the metal-free centrifuge tube to ensure no sample remained in the tube. ICP-MS samples of E. coli and corresponding controls were prepared similarly using LB and AM media (72). Samples were stored at 4°C until analysis by ICP-MS. All flasks, centrifuge tubes, containers, Teflon tubes, and pipet tips are plastic and were acid-washed before use.

Cell samples were analyzed with an Agilent 8800 triple-quadrupole ICP-MS instrument (ICP-QQQ/Agilent Technologies, Japan) equipped with an SPS 4 autosampler and using 2% trace metal HNO_3_ as the blank. The following settings were fixed for analysis: cell entrance, −50 V; cell exit, −70 V; plate bias, −70 V; octP bias, −18 V; collision cell helium flow, 4.3 mL/min. Optimal voltages for Extract 2, Omega Bias, Omega Lens, OctP RF, and Deflect were determined via auto tune with 1 ppb instrument tuning solution before each sample set was analyzed. Samples were introduced by a peristaltic pump with 0.5-mm-internal-diameter tubing through a MicroMist borosilicate glass nebulizer (Agilent). Samples were initially taken up at 0.3 rps for 50 seconds then stabilized for 15 seconds at 0.1 rps. Samples were analyzed in spectrum mode at 0.1 rps and three replicates of 100 sweeps were performed for each element analyzed. Sampling probe and tubing were rinsed for 90 seconds at 0.3 rps with 2% trace metal HNO_3_ after every sample. Agilent Mass Hunter Workstation was used for data acquisition and analysis. ICP-MS calibration standards were prepared in the same manner as ICP-OES standard, and contain 1,000, 750, 500, 250, 100, 40, 25, 16, 10, 5, 2.5, 1, and 0.5 ppb for each metal.

Metal content was quantified by converting metal concentrations in ppb to atoms/CFU using CFU values calculated from serial dilutions corresponding to each sample (described above). Metal contents of cells were measured by ICP-MS for Ca, V, Cr, Mn, Fe, Co, Ni, Cu, Zn, Se, Mo, and Cd. ICP-MS data are combined from three biological replicates that were each measured with three technical triplicates, with the means and standard deviations graphed. Significance was determined by one-way ANOVA with Tukey multiple comparison test. Statistical significance is indicated as follows: *P ≤ 0.05; **P ≤ 0.01; ***P ≤ 0.001; ****P ≤ 0.0001; n.s., not significant.

### Overnight culture growth curves.

*Lactobacillus* species were first grown on MRS agar and then grown 18 h to 20 h in MRS broth. Overnight cultures were diluted to OD_600_ of 5 in fresh MRS medium or washed with CDM medium then diluted to OD_600_ of 5 in fresh CDM. Growth kinetics studies in MRS broth were conducted by inoculating 200 μL of MRS broth containing varied concentrations of ZnSO_4_ (0 to 500 or 0 to 1,000 μM), MnCl_2_ (0 to 250 μM), or FeSO_4_ (0 to 150 μM) with 2 μL of an overnight *Lactobacillus* culture and incubated at 37°C in an atmosphere of 5% CO_2_ with shaking at 200 rpm. For growth kinetics studies in CDM or CDM supplemented with Wolfe solutions using washed cultures for inoculation, 200 μL of CDM medium or supplemented (with 1% Wolfe solution) CDM medium and varied concentrations of ZnSO_4_ (0 to 500 or 1,000 μM), MnCl_2_ (0 to 250μM), or FeSO_4_ (0 to 150 μM) were inoculated with 2 μL of washed culture and incubated with continuous shaking (aerobic/5% CO_2_, 37°C). CDM medium used for manganese studies is prepared without added MnCl_2_ (Table S1). The Wolfe solution for each set of metal studies was described above in the Media and supplements section. Growth kinetics experiments for E. coli were performed similarly using LB and AM media (aerobic, 37°C). For each sample, the OD_600_ was recorded every 30 min until growth slowed and declined, using the corresponding fresh medium as the blank. A control was carried out in parallel for each condition in the absence of added bacteria. Each condition was performed with at least three biological replicates, each with two to three technical replicates. Representative growth curves shown are from one biological replicate to clearly demonstrate the effect of metal on each strain. *Lactobacillus* species growth rates can vary between biological replicates but all metal-dependent trends observed remained consistent between biological replicates. Each representative growth curve is accompanied with graphs showing calculated lag time, growth rate, and max OD (Fig. 5 and supplementary material) from three biological replicates to demonstrate the consistency of metal effects. Lag time, growth rate, and max OD represent the mean ± SEM of three biological replicates, each with ≥2 technical replicates. These growth parameters were calculated using nonlinear regression curve fitting to a four-parameter logistic equation with GraphPad Prism 9 software (GraphPad software, CA) (130–132).

### Subculture growth curves.

*Lactobacilli* starter cultures were grown overnight (18 h to 20 h) in MRS from a fresh (1 week or less) MRS/agar streak plate (Scheme S1, day 1) as for the overnight growth curves. On day 2, MRS subcultures were prepared by inoculating 75 μL overnight starter culture in 5 mL MRS and growing 24 h. Overnight starter cultures were washed with CDM before inoculation (using 200 μL washed culture) into fresh CDM (5 mL) and grown for 25 h for all CDM subcultures. Mucin subcultures were prepared using 5 mL CDM containing 0.1% mucin. Zinc, manganese, and iron subcultures were prepared similarly using CDM containing 100 μM ZnSO_4_, 50 μM MnCl_2_, and 25 μM FeSO_4_, respectively (Scheme S1). E. coli subcultures for studying the effects of zinc were prepared similarly using corresponding LB and AM media. Growth kinetics studies were then conducted (day 3) by inoculating 2 μL of each subculture into the corresponding experimental medium (200 μL), MRS or supplemented CDM, each with varied concentrations of ZnSO_4_ (0 to 500 or 0 to 1,000 μM), MnCl_2_ (0 to 250 μM), or FeSO_4_ (0 to 150 μM). Data were collected and presented as described above for overnight growth curves. Each condition was performed with three biological replicates.

### Bright-field microscopy of lactobacilli.

The OD_600_ of each microscope sample was first adjusted to 5, then 2 μL of culture was pipetted onto 3% agarose deposited on a glass slide and secured with a glass coverslip. Bright-field images were captured on an Olympus IX83 inverted microscope equipped with a scientific CMOS camera (Photometric Prime 95B). The samples were imaged using a 100X TIRF oil immersion objective (Olympus UApoN 100X TIRF) with a 2X coded intermediate magnification changer (IX3-CAS). All observed images were magnified by 200X and captured with CellSense imaging software.
