# Supplementary material for: Differential Effects of Transition Metals on Growth and Metal Uptake for Two Distinct Lactobacillus Species
Source: Microbiol Spectr. 2022 Jan 26;10(1):e01006-21. doi: 10.1128/spectrum.01006-21 (PMC8791193; doi:10.1128/spectrum.01006-21)
Supplement: SUPPLEMENTAL FILE 1 — Supplemental material. Download Spectrum01006-21_Supplemental_Material-updated-1-18-2022.pdf, PDF file, 1.8 MB [file spectrum01006-21_supplemental_material-updated-1-18-2022.pdf]

Supplemental Material

Differential effects of transition metals on growth and metal uptake for two distinct *Lactobacillus* species

Uyen Huynh[a], Muxin Qiao[a], John King[a], Brittany Trinh[a], Juventino Valdez[a], Marium Haq[a], Melissa L. Zastrow[a]

[a]Department of Chemistry, University of Houston, Houston, TX, USA

Table of contents

Scheme S1. Subculture procedure for growth kinetics studies S-2
Table S1. Components of chemically defined minimal (CDM) medium S-3
Table S2. Metal content of MRS and CDM medium as quantified by ICP-OES S-4
Table S3. Metal content of *L. plantarum*, *L. acidophilus*, and *E. coli* as quantified by ICP-MS S-5
Table S4. Growth parameters of subculture growth for *L. plantarum* ATCC 14917 S-6
Table S5. Growth parameters of subculture growth for *L. acidophilus* ATCC 4356 S-7
Figure S1. Metal content of *E. coli* BW25113 S-8
Figure S2. Effect of zinc on the growth of *Lactobacillus* species and *E. coli* in rich medium S-9
Figure S3. Effect of trace mineral supplement on zinc-dependent growth of *L. plantarum* and *L. acidophilus* S-10
Figure S4. Effect of zinc gluconate and zinc sulfate on the growth parameters of *L. plantarum* grown in MRS and CDM media. S-11
Figure S5. Effect of zinc on subculture growth for *L. plantarum* S-12
Figure S6. Effect of zinc on subculture growth for *L. acidophilus* S-13
Figure S7. Effect of manganese on subculture growth for *L. plantarum* S-14
Figure S8. Effect of manganese on subculture growth for *L. acidophilus* S-15
Figure S9. Effect of iron on subculture growth for *L. plantarum* S-16
Figure S10. Effect of iron on subculture growth for *L. acidophilus* S-17
Figure S11. Bright field images of *Lactobacilli* grown in different subculture media S-18

Scheme S1. Subculture procedure for growth kinetics studies.[a]

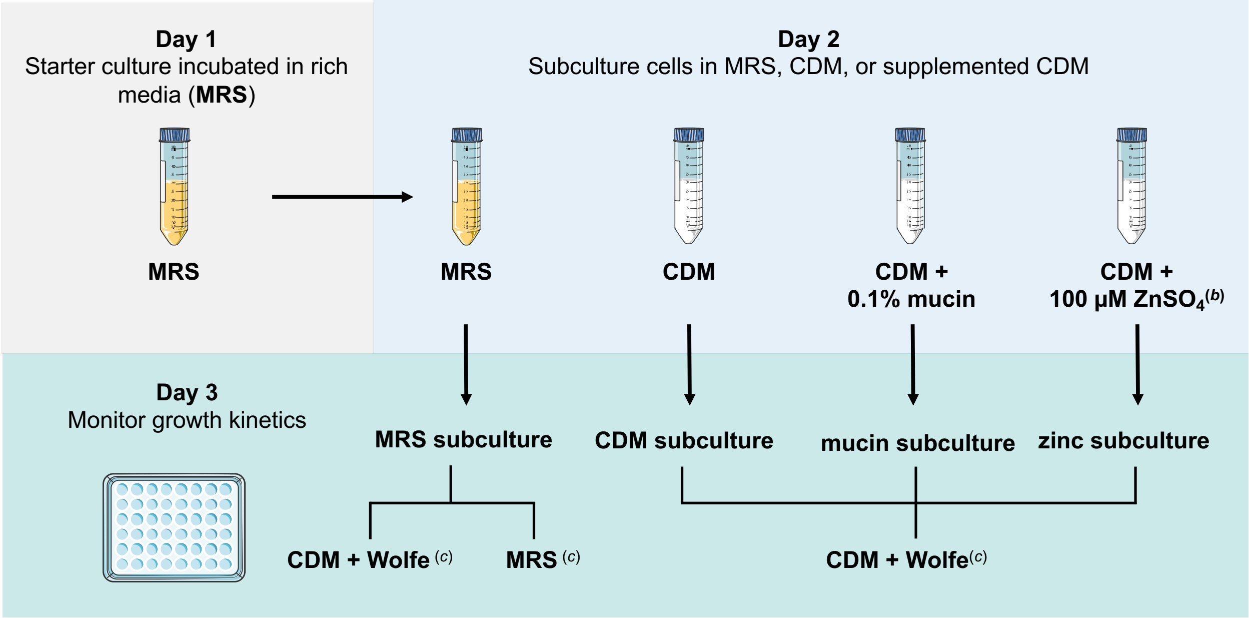

(a) Images adapted from Servier Medical Art by Servier (smart.servier.com).
(b) 50 μM $MnCl_2$ and 25 μM $FeSO_4$ used for manganese and iron studies, respectively.
(c) Fresh medium with varied metal concentrations. CDM (see Table S1 and Methods) used for zinc studies is supplemented with 1% Wolfe trace mineral solution containing all metals except zinc. CDM used for manganese studies is prepared without added manganese and supplemented with 1% Wolfe trace mineral solution containing all metals except manganese (see Methods). CDM used for iron studies is supplemented with 1% Wolfe trace mineral solution containing all metals except iron (see Methods).

Table S1. Components of chemically defined minimal (CDM) medium.[a]

Component		Final concentration in media (g/L)	(mmol/L)
**Solution 1**	$MgSO_4 \cdot 7H_2O$	0.150	1.249
	$MnCl_2$[b]	0.020	0.159
**Solution 2**[c]	Adenine (vitamin B4)	0.010	0.074
	Biotin (Vitamin B7)	0.003	0.012
	Nicotinic acid (Niacin /Vitamin B3)	0.001	0.008
	D-aminobenzoic acid (para or 4-)	0.010	0.073
	Pyridoxal HCl (vitamin B6 HCl)	0.002	0.010
	Thiamine HCl (vitamin B1)	0.001	0.003
**Solution 3**	Glutamic acid	0.500	3.398
	Aspartic acid	0.420	3.156
	Glycine	0.175	2.331
	Histidine	0.150	0.716
	Isoleucine	0.210	1.601
	Leucine	0.475	3.621
	DL-Alanine	0.240	2.694
	Arginine	0.125	0.593
	Lysine	0.440	2.409
	Phenylalanine	0.275	1.665
	Proline	0.675	5.863
	Serine	0.340	3.235
	Threonine	0.225	1.889
	Tyrosine	0.250	1.380
	Valine	0.325	2.774
	Cysteine-HCl	0.130	0.740
	Methionine	0.125	0.838
	Tryptophan	0.050	0.245
**Solution 4**[c]	Uracil	0.006	0.054
	Guanine	0.005	0.033
	Xanthine	0.005	0.033
**Solution 5**	K_2HPO_4	4.560	26.177
	$(NH_4)_2SO_4$	2.000	15.136
**Solution 6**	D-glucose	10.000	55.506
**Solution 7**	Folic acid (vitamin B11)	0.001	0.002
**Solution 8**[c]	Riboflavin (vitamin B2)	0.001	0.003
**Solution 9**	Ca-(D)-(+)-pantothenate (vitamin B5)	0.0005	0.00209846
**Solution 10**[d]	Cobalamin (vitamin B12)	0.000001	4.1969E-06

[a] All components are either treated with Chelex or are trace metal grade (see Methods).
[b] $MnCl_2$ was omitted for CDM medium used in the manganese studies.
[c] 10 M NaOH was added to solution 2, 4, 8 in small aliquots until solution became clear.
[d] Solution 10 was used immediately upon preparation.

Table S2. Metal content of MRS and CDM medium as quantified by ICP-OES.

	MRS (μM)	CDM (μM)
**Ca**	800 ± 200	1.0 ± 0.2
**V**	0.9 ± 0.2	0.003 ± 0.001
**Cr**	0.4 ± 0.1	0.15 ± 0.06
**Mn**	190 ± 60	108 ± 9[a]
**Fe**	11 ± 3	0.07 ± 0.02
**Co**	0.15 ± 0.08	Not detected
**Ni**	0.21 ± 0.07	Not detected
**Cu**	0.18 ± 0.09	Not detected
**Zn**	20 ± 5	0.020 ± 0.002
**Se**	0.9 ± 0.1	0.15 ± 0.03
**Mo**	0.11 ± 0.03	0.002 ± 0.001
**Cd**	Not detected	0.0004 ± 0.0002

[a] [Mn^{2+}] detected in CDM medium is from $MnCl_2$ supplemented in CDM medium (Table S1), which was used in zinc and iron studies as described in Methods. CDM medium used for manganese studies is prepared without the $MnCl_2$ supplement and has a likely Mn^{2+} concentration <0.01 μM (based on ICP-OES data for mQ water used to prepare CDM medium, see Methods).

Table S3. Metal content of *L. plantarum*, *L. acidophilus*, and *E. coli* as quantified by ICP-MS.

	MRS		CDM	
	(atoms/CFU)	(nmol/CFU)	(atoms/CFU)	(nmol/CFU)
***L. plantarum* ATCC 14917**				
**Ca**	6.6(±4.2) x 10^{5}	1.1(±0.7) x 10^{-9}	2.1(±1.3) x 10^{5}	3.5(±2.2) x 10^{-10}
**V**	9.2(±7.4) x 10^{3}	1.5(±1.2) x 10^{-11}	9.0(±8.4) x 10^{3}	1.5(±1.4) x 10^{-11}
**Cr**	8.3(±5.6) x 10^{4}	1.4(±0.9) x 10^{-10}	1.1(±0.8) x 10^{4}	1.9(±1.3) x 10^{-11}
**Mn**	2.5 (±0.1) x 10^{7}	4.1(±1.7) x 10^{-8}	5.5(±2.8) x 10^{7}	9.2(±4.7) x 10^{-8}
**Fe**	4.7(±4.4) x 10^{5}	7.8(±7.4) x 10^{-10}	1.4(±1.3) x 10^{5}	2.4(±2.2) x 10^{-10}
**Co**	7.7(±5.4) x 10^{3}	1.3(±0.9) x 10^{-11}	8.9(±7.1) x 10^{3}	1.5(±1.2) x 10^{-11}
**Ni**	6.2(±4.5) x 10^{4}	1.0(±0.7) x 10^{-10}	1.8(±1.8) x 10^{4}	3.0(±2.9) x 10^{-11}
**Cu**	1.1(±0.4) x 10^{5}	1.8(±0.6) x 10^{-10}	1.1(±0.8) x 10^{4}	1.8(±1.3) x 10^{-11}
**Zn**	2.8(±0.5) x10^{5}	4.7(±0.9) x 10^{-10}	1.6(±0.6) x 10^{5}	2.6(±1.0) x 10^{-10}
**Se**	7.3(±5.3) x 10^{3}	1.2(±0.9) x 10^{-11}	6.7(±5.7) x 10^{3}	1.1(±0.9) x 10^{-11}
**Mo**	6.1(±3.5) x 10^{3}	1.0(±0.6) x 10^{-11}	6.1(±3.9) x 10^{3}	1.0(±0.6) x 10^{-11}
**Cd**	5.6(±3.5) x 10^{3}	9.3(±5.8) x 10^{-12}	1.6(±1.4) x 10^{5}	2.7(±2.3) x 10^{-10}
***L. acidophilus* ATCC 4356**				
**Ca**	5.0(±1.2) x 10^{5}	8.4(±2.0) x 10^{-10}	3.2(±2.3) x 10^{5}	5.4(±3.8) x 10^{-10}
**V**	1.0(±0.8) x 10^{4}	1.7(±1.3) x 10^{-11}	1.05(±1.04) x 10^{4}	1.8(±1.7) x 10^{-11}
**Cr**	1.8(±0.7) x 10^{4}	3.0(±1.1) x 10^{-11}	2.6(±2.0) x 10^{4}	4.3(±3.4) x 10^{-11}
**Mn**	2.1(±1.9) x 10^{7}	3.5(±3.2) x 10^{-8}	1.3(±1.1) x 10^{8}	2.1(±1.8) x 10^{-7}
**Fe**	1.3(±0.6) x 10^{5}	2.2(±1.0) x 10^{-10}	1.4(±0.6) x 10^{5}	2.3(±1.0) x 10^{-10}
**Co**	7.6(±5.3) x 10^{3}	1.3(±0.9) x 10^{-11}	1.2(±0.6) x 10^{4}	2.1(±1.0) x 10^{-11}
**Ni**	1.1(±0.6) x 10^{4}	1.8(±1.0) x 10^{-11}	1.5(±0.9) x 10^{3}	2.4(±1.6) x 10^{-11}
**Cu**	1.8(±0.9) x 10^{4}	3.0(±1.4) x 10^{-11}	8.1(±4.6) x 10^{3}	1.3(±0.8) x 10^{-11}
**Zn**	2.0(±1.6) x 10^{5}	3.4(±2.7) x 10^{-10}	5.0(±2.1) x 10^{5}	8.3(±3.6) x 10^{-10}
**Se**	9.1(±3.6) x 10^{3}	1.5(±0.6) x 10^{-11}	8.8(±5.1) x 10^{3}	1.5(±0.8) x 10^{-11}
**Mo**	5.1(±2.5) x 10^{3}	8.4(±4.2) x 10^{-12}	8.6(±2.4) x 10^{3}	1.4(±0.4) x 10^{-11}
**Cd**	6.0(±2.2) x 10^{3}	1.0(±0.4) x 10^{-11}	7.6(±2.1) x 10^{3}	1.3(±0.3) x 10^{-11}
***E. coli* BW 25113**				
**Ca**	1.8(±1.4) x 10^{5}	2.9(±2.3) x 10^{-10}	1.6(±1.3) x 10^{5}	2.6(±2.1) x 10^{-10}
**V**	5.3(±3.6) x 10^{2}	8.3(±5.9) x 10^{-13}	3.0(±2.6) x 10^{2}	4.9(±4.4) x 10^{-13}
**Cr**	1.8(±1.7) x 10^{4}	3.0(±2.8) x 10^{-11}	8.7(±7.8) x 10^{3}	1.4(±1.3) x 10^{-11}
**Mn**	3.9(±2.9) x 10^{3}	6.5(±4.8) x 10^{-12}	9.0(±5.4) x 10^{2}	1.5(±0.9) x 10^{-12}
**Fe**	6.6(±3.0) x 10^{5}	1.1(±0.5) x 10^{-9}	6.1(±3.7) x 10^{5}	1.0(±0.6) x 10^{-9}
**Co**	9.9(±5.7) x 10	1.6(±1.0) x 10^{-13}	4.2(±1.9) x 10	6.9(±3.2) x 10^{-14}
**Ni**	2.5(±1.5) x 10^{3}	4.2(±2.5) x 10^{-12}	8.8(±4.4) x 10^{2}	1.5(±0.7) x 10^{-12}
**Cu**	4.1(±3.5) x 10^{4}	6.8(±5.9) x 10^{-11}	4.6(±4.4) x 10^{3}	7.7(±7.4) x 10^{-12}
**Zn**	3.6(±1.1) x 10^{4}	5.9(±1.8) x 10^{-11}	1.5(±0.9) x 10^{4}	2.4(±1.4) x 10^{-11}
**Se**	1.6(±1.2) x 10^{3}	2.7(±1.9) x 10^{-12}	5.4(±2.4) x 10^{2}	8.9(±4.1) x 10^{-13}
**Mo**	2.5(±0.7) x 10^{3}	4.1(±1.1) x 10^{-12}	1.3(±0.4) x 10^{3}	2.2(±0.6) x 10^{-12}

Table S4. Growth parameters of subculture growth for *L. plantarum* ATCC 14917 in CDM supplemented with 1% Wolfe and varied concentration of zinc, manganese, and iron.[a]

***L. plantarum* ATCC 14917**							
**Subculture**	**[Zn^{2+}]**	**0 μM**	**50 μM**	**75 μM**	**100 μM**	**250 μM**	**500 μM**
**MRS**	**Lag time (h)**	4.1±0.4	4.0±0.1	3.8±0.1	3.8±0.1	3.9±0.2	4.8±0.2
	**Growth rate (h^{-1})**	0.24±0.02	0.25±0.01	0.26±0.01	0.26±0.01	0.25±0.01	0.21±0.01
	**Max OD**	1.4±0.05	1.62±0.02	1.62±0.02	1.64±0.02	1.59±0.02	1.61±0.03
**CDM**	**Lag time (h)**	5.9±0.2	4.1±0.2	3.9±0.2	4.0±0.1	4.1±0.1	5.3±0.2
	**Growth rate (h^{-1})**	0.17±0.01	0.25±0.01	0.26±0.01	0.25±0.01	0.24±0.01	0.19±0.01
	**Max OD**	1.51±0.05	1.69±0.04	1.64±0.03	1.65±0.03	1.67±0.03	1.8±0.06
**Mucin**	**Lag time (h)**	4.2±0.3	4.1±0.3	4±0.3	4.2±0.3	4.5±0.3	5.5±0.4
	**Growth rate (h^{-1})**	0.24±0.01	0.25±0.02	0.25±0.02	0.24±0.01	0.22±0.01	0.18±0.01
	**Max OD**	1.36±0.04	1.58±0.05	1.58±0.05	1.57±0.05	1.57±0.05	1.59±0.08
**Zinc**	**Lag time (h)**	4.6±0.3	4.2±0.2	4.3±0.2	4.3±0.2	4.8±0.2	5.9±0.3
	**Growth rate (h^{-1})**	0.22±0.01	0.24±0.01	0.23±0.01	0.23±0.01	0.21±0.01	0.17±0.01
	**Max OD**	1.39±0.05	1.57±0.04	1.6±0.04	1.59±0.05	1.64±0.06	1.75±0.1
**Subculture**	**[Mn^{2+}]**	**0 μM**	**25 μM**	**50μM**	**75 μM**	**100 μM**	**250 μM**
**MRS**	**Lag time (h)**	3.9±0.2	3.5±0.1	3.8±0.2	3.6±0.1	3.0±0.1	3.2±0.1
	**Growth rate (h^{-1})**	0.26±0.01	0.29±0.01	0.26±0.01	0.28±0.01	0.33±0.02	0.31±0.01
	**Max OD**	1.27±0.02	1.52±0.02	1.46±0.03	1.67±0.02	1.83±0.02	1.87±0.02
**CDM**	**Lag time (h)**	3.4±0.1	3.9±0.1	3.7±0.1	3.5±0.1	3.3±0.1	3.3±0.1
	**Growth rate (h^{-1})**	0.29±0.01	0.26±0.01	0.27±0.01	0.29±0.01	0.3±0.01	0.3±0.01
	**Max OD**	1.29±0.01	1.42±0.01	1.3±0.01	1.53±0.01	1.62±0.01	1.8±0.02
**Mucin**	**Lag time (h)**	4.3±0.2	3.7±0.2	3.9±0.1	3.6±0.1	3.8±0.1	3.1±0.1
	**Growth rate (h^{-1})**	0.23±0.01	0.27±0.01	0.25±0.01	0.28±0.01	0.26±0.01	0.32±0.01
	**Max OD**	1.3±0.03	1.37±0.02	1.33±0.02	1.52±0.02	1.65±0.03	1.93±0.03
**Manganese**	**Lag time (h)**	4.1±0.2	3.8±0.1	3.9±0.1	3.5±0.2	3.5±0.1	3.8±0.1
	**Growth rate (h^{-1})**	0.25±0.01	0.26±0.01	0.26±0.01	0.28±0.01	0.28±0.01	0.26±0.01
	**Max OD**	1.34±0.02	1.37±0.02	1.38±0.02	1.56±0.03	1.69±0.02	1.84±0.03
**Subculture**	**[Fe^{2+}]**	**0 μM**	**10 μM**	**25μM**	**50μM**	**100 μM**	**150 μM**
**MRS**	**Lag time (h)**	4.4±0.2	4.3±0.2	4.1±0.2	4.1±0.2	3.1±0.3	2.9±0.3
	**Growth rate (h^{-1})**	0.23±0.01	0.23±0.01	0.24±0.01	0.25±0.01	0.33±0.03	0.35±0.03
	**Max OD**	1.6±0.04	1.56±0.04	1.57±0.04	1.55±0.04	1.8±0.05	1.78±0.05
**CDM**	**Lag time (h)**	4.2±0.1	4.2±0.1	4.2±0.1	4.2±0.1	4.1±0.1	3.8±0.1
	**Growth rate (h^{-1})**	0.24±0.01	0.24±0.01	0.24±0.01	0.24±0.01	0.25±0.01	0.26±0.01
	**Max OD**	1.52±0.01	1.47±0.01	1.47±0.01	1.47±0.01	1.47±0.01	1.47±0.01
**Mucin**	**Lag time (h)**	4.4±0.2	4.2±0.1	4.2±0.2	4.1±0.2	4.1±0.2	4.1±0.2
	**Growth rate (h^{-1})**	0.23±0.01	0.24±0.01	0.24±0.01	0.24±0.01	0.25±0.01	0.24±0.01
	**Max OD**	1.48±0.02	1.44±0.01	1.44±0.02	1.42±0.01	1.42±0.01	1.43±0.01
**Iron**	**Lag time (h)**	4.2±0.1	4.0±0.1	4.0±0.1	3.9±0.1	3.9±0.1	4.0±0.1
	**Growth rate (h^{-1})**	0.24±0.01	0.25±0.01	0.25±0.01	0.26±0.01	0.25±0.01	0.25±0.01
	**Max OD**	1.49±0.01	1.46±0.01	1.45±0.01	1.45±0.01	1.45±0.01	1.43±0.01

[a] Lag time, growth rate, and max OD represent the mean ± SEM of three biological replicates, each with ≥2 technical replicates. Growth parameters were calculated using nonlinear regression curve fitting to a 4-parameter Logistic equation with GraphPad Prism 9 software.

Table S5. Subculture growth parameters for *L. acidophilus* ATCC 4356 in CDM supplemented with 1% Wolfe and varied concentrations of zinc, manganese, and iron.[a]

L. acidophilus ATCC 4356							
**Subculture**	**[Zn^{2+}]**	**0 μM**	**50 μM**	**75 μM**	**100 μM**	**250 μM**	**500 μM**
**MRS**	**Lag time (h)**	3.4±0.1	3.3±0.1	3.3±0.1	3.3±0.1	3.4±0.1	4.1±0.1
	**Growth rate (h^{-1})**	0.29±0.01	0.30±0.01	0.31±0.01	0.30±0.01	0.30±0.01	0.24±0.01
	**Max OD**	2.07±0.01	2.08±0.01	2.1±0.01	2.1±0.01	2.11±0.01	2.12±0.01
**CDM**	**Lag time (h)**	3.8±0.1	3.5±0.1	3.5±0.1	3.7±0.2	4.0±0.2	4.8±0.3
	**Growth rate (h^{-1})**	0.26±0.01	0.29±0.01	0.29±0.01	0.27±0.01	0.25±0.01	0.21±0.01
	**Max OD**	1.96±0.02	1.98±0.02	1.98±0.02	1.92±0.02	1.84±0.03	1.86±0.04
**Mucin**	**Lag time (h)**	3.6±0.1	3.5±0.1	3.7±0.1	3.9±0.1	4.0±0.1	4.7±0.1
	**Growth rate (h^{-1})**	0.28±0.01	0.29±0.01	0.27±0.01	0.26±0.01	0.25±0.01	0.21±0.01
	**Max OD**	2.04±0.01	2.13±0.01	2.11±0.01	2.06±0.01	2.03±0.01	2.02±0.02
**Zinc**	**Lag time (h)**	3.2±0.1	3.0±0.1	3.1±0.1	3.4±0.1	3.5±0.1	4.3±0.3
	**Growth rate (h^{-1})**	0.31±0.01	0.33±0.01	0.32±0.01	0.3±0.01	0.29±0.01	0.23±0.01
	**Max OD**	1.88±0.01	1.9±0.01	1.89±0.01	1.87±0.01	1.75±0.02	1.7±0.03
**Subculture**	**[Mn^{2+}]**	**0 μM**	**25 μM**	**50 μM**	**75 μM**	**100 μM**	**250 μM**
**MRS**	**Lag time (h)**	3.5±0.2	3.5±0.2	3.3±0.2	3.5±0.2	3.6±0.2	3.7±0.2
	**Growth rate (h^{-1})**	0.29±0.02	0.28±0.01	0.3±0.02	0.29±0.02	0.28±0.02	0.27±0.02
	**Max OD**	1.61±0.02	2.03±0.02	2.05±0.02	2.09±0.02	2.12±0.03	2.17±0.03
**CDM**	**Lag time (h)**	3.9±0.2	3.5±0.1	3.1±0.1	2.8±0.1	2.9±0.1	3.0±0.1
	**Growth rate (h^{-1})**	0.26±0.02	0.28±0.01	0.32±0.01	0.35±0.01	0.35±0.01	0.34±0.01
	**Max OD**	1.55±0.02	2.07±0.01	1.97±0.01	2.0±0.01	2.03±0.01	2.05±0.01
**Mucin**	**Lag time (h)**	3.8±0.2	3.0±0.1	3.1±0.1	3.1±0.1	3.1±0.1	3.4±0.1
	**Growth rate (h^{-1})**	0.26±0.01	0.33±0.01	0.32±0.01	0.32±0.01	0.32±0.01	0.29±0.01
	**Max OD**	1.54±0.02	2.06±0.01	2.03±0.01	2.03±0.01	2.05±0.01	2.13±0.01
**Manganese**	**Lag time (h)**	3.7±0.2	2.8±0.1	2.8±0.1	2.4±0.1	2.5±0.1	2.8±0.1
	**Growth rate (h^{-1})**	0.27±0.01	0.36±0.01	0.36±0.01	0.41±0.01	0.4±0.01	0.36±0.01
	**Max OD**	1.66±0.02	2.04±0.01	1.94±0.01	1.97±0.01	2.01±0.01	2.01±0.01
**Subculture**	**[Fe^{2+}]**	**0 μM**	**10 μM**	**25μM**	**50μM**	**100 μM**	**150 μM**
**MRS**	**Lag time (h)**	4.0±0.1	4.2±0.1	3.6±0.1	3.7±0.1	2.8±0.1	2.8±0.1
	**Growth rate (h^{-1})**	0.25±0.01	0.24±0.01	0.28±0.01	0.27±0.01	0.36±0.01	0.36±0.01
	**Max OD**	2.11±0.02	2.1±0.02	2.12±0.01	2.11±0.01	2.14±0.01	2.13±0.01
**CDM**	**Lag time (h)**	4.0±0.1	4.0±0.1	4.2±0.1	4.4±0.1	4.4±0.1	4.4±0.1
	**Growth rate (h^{-1})**	0.25±0.01	0.25±0.01	0.24±0.01	0.23±0.01	0.23±0.01	0.23±0.01
	**Max OD**	2.09±0.02	2.08±0.01	2.08±0.01	2.06±0.01	2.04±0.01	1.99±0.02
**Mucin**	**Lag time (h)**	3.6±0.1	3.7±0.1	3.9±0.1	4.0±0.1	4.1±0.1	4.2±0.1
	**Growth rate (h^{-1})**	0.28±0.01	0.27±0.01	0.26±0.01	0.25±0.01	0.25±0.01	0.24±0.01
	**Max OD**	2.1±0.01	2.08±0.01	2.07±0.01	2.04±0.01	2.01±0.01	2±0.01
**Iron**	**Lag time (h)**	3.7±0.1	3.7±0.1	3.9±0.1	3.9±0.1	4.1±0.1	4.1±0.1
	**Growth rate (h^{-1})**	0.27±0.01	0.27±0.01	0.26±0.01	0.25±0.01	0.24±0.01	0.25±0.01
	**Max OD**	2.08±0.02	2.08±0.02	2.08±0.02	2.07±0.01	2.05±0.01	2.06±0.01

[a] Lag time, growth rate, and max OD represent the mean ± SEM of three biological replicates, each with ≥2 technical replicates. Growth parameters were calculated using nonlinear regression curve fitting to a 4-parameter Logistic equation with GraphPad Prism 9 software.

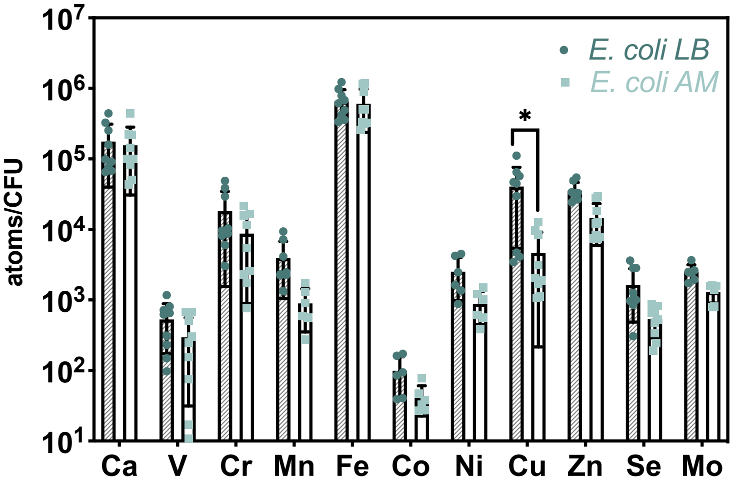

Figure S1. Metal content of *E. coli* BW25113 mid-log phase cells grown in rich (LB) and metal-limited A minimal medium as measured by ICP-MS. Error bars are SD of 3 biological replicates, each with ≥2 technical replicates. *$p \leq 0.05$ as determined by one-way ANOVA with Tukey multiple comparison test.

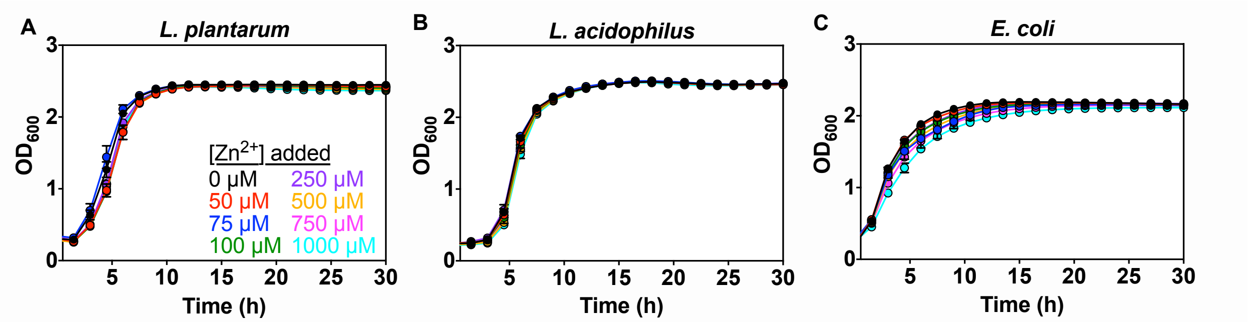

Figure S2. Effect of zinc on the growth of *Lactobacillus* species (A-B) in rich medium (MRS) and *E. coli* (C) in rich medium (LB). Representative growth curves are shown with error bars as SD from one representative biological replicate, each with ≥2 technical replicates.

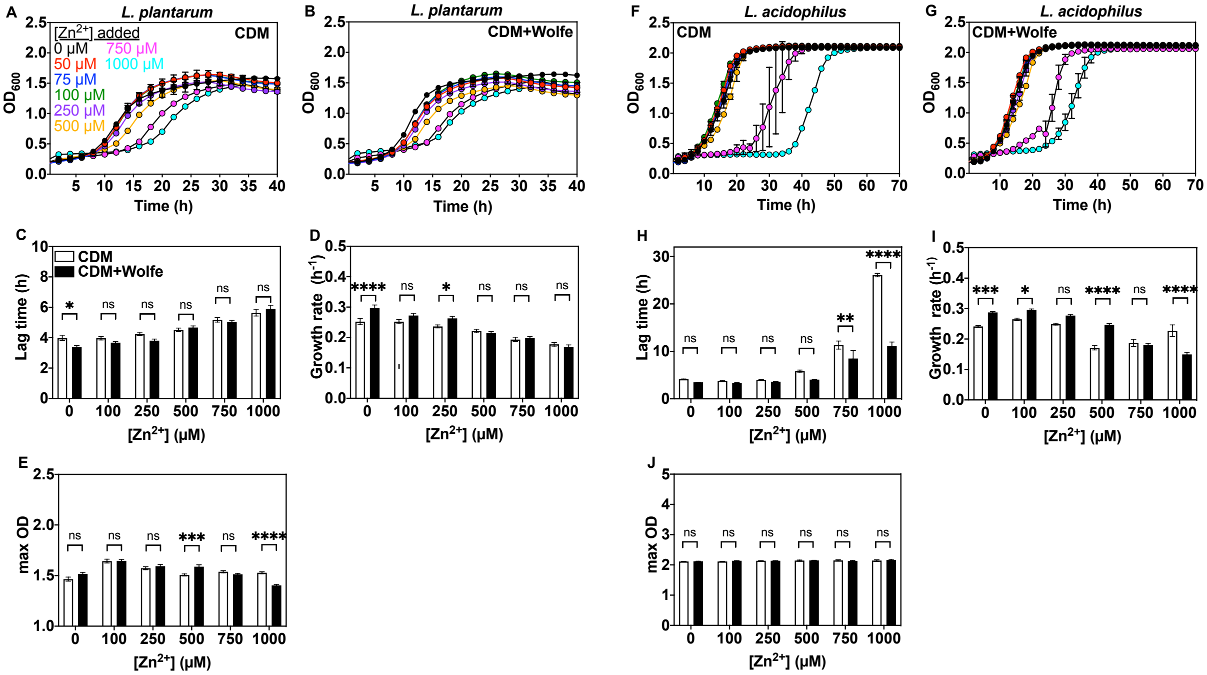

Figure S3. Zinc dependence of the growth parameters of *L. plantarum* ATCC 14917 (A-E) and *L. acidophilus* ATCC 4356 (F-J) in metal limited CDM medium with and without 1% Wolfe trace mineral supplement as measured by OD_{600}. Lag time, growth rate, and max OD beneath each set of representative growth curves represent the mean ± SEM of three biological replicates, each with ≥2 technical replicates. Growth parameters were calculated using nonlinear regression curve fitting to a 4-parameter Logistic equation with GraphPad Prism 9 software. ns (not significant), *p≤0.05, **p≤0.01, ***p≤0.001, ****p≤0.0001 as determined by two-way ANOVA with Sidak multiple comparison test. The supplement does not affect lag time, and max OD for either species, except at high added Zn^{2+} concentrations, where the max OD for *L. plantarum* was suppressed (1000 μM Zn^{2+}) and the lag time for *L. acidophilus* was shortened (750-1000 μM Zn^{2+}) in the presence of the trace mineral supplement. The 1% Wolfe trace mineral supplement promotes growth rate significantly at 0 μM Zn^{2+} for *L. plantarum*, and slightly at 100-250 μM Zn^{2+}. The supplement generally increased the growth rate for *L. acidophilus* at 0-500 μM Zn^{2+} but repressed it at 1000 μM Zn^{2+}.

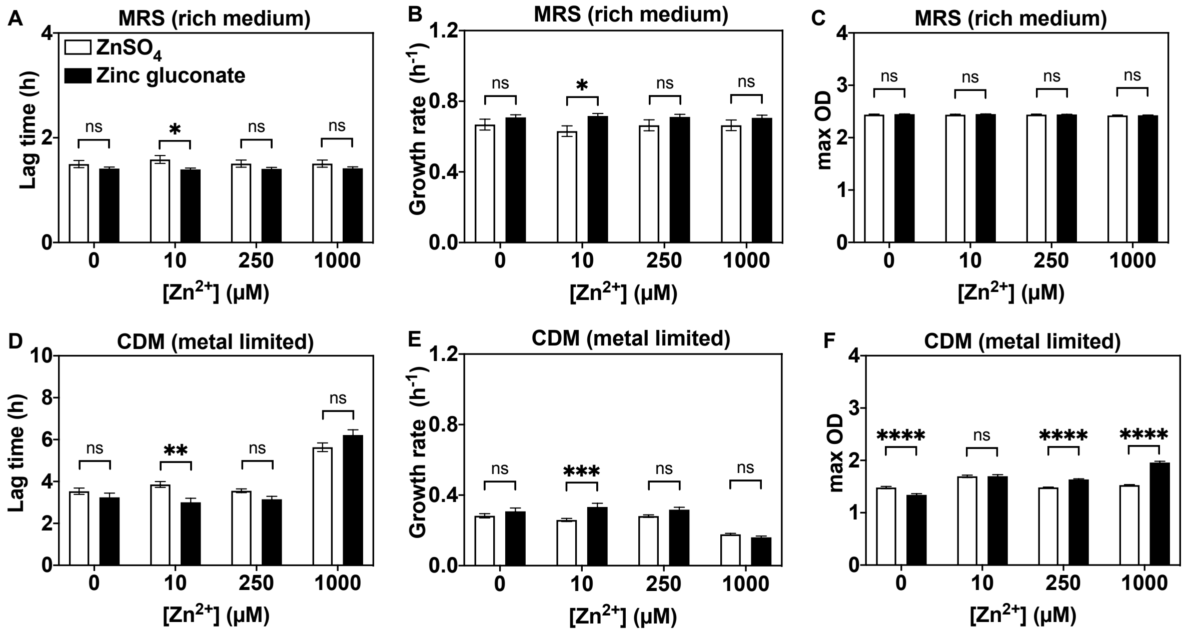

Figure S4. Effect of zinc gluconate and zinc sulfate on the growth parameters of *L. plantarum* grown in MRS and CDM media. Lag time, growth rate, and max OD represent the mean ± SEM of three biological replicates, each with ≥2 technical replicates. Growth parameters were calculated using nonlinear regression curve fitting to a 4-parameter Logistic equation with GraphPad Prism 9 software. ns (not significant), $^{*}p \leq 0.05$, $^{**}p \leq 0.01$, $^{***}p \leq 0.001$, $^{****}p \leq 0.0001$ as determined by two-way ANOVA with Sidak multiple comparison test.

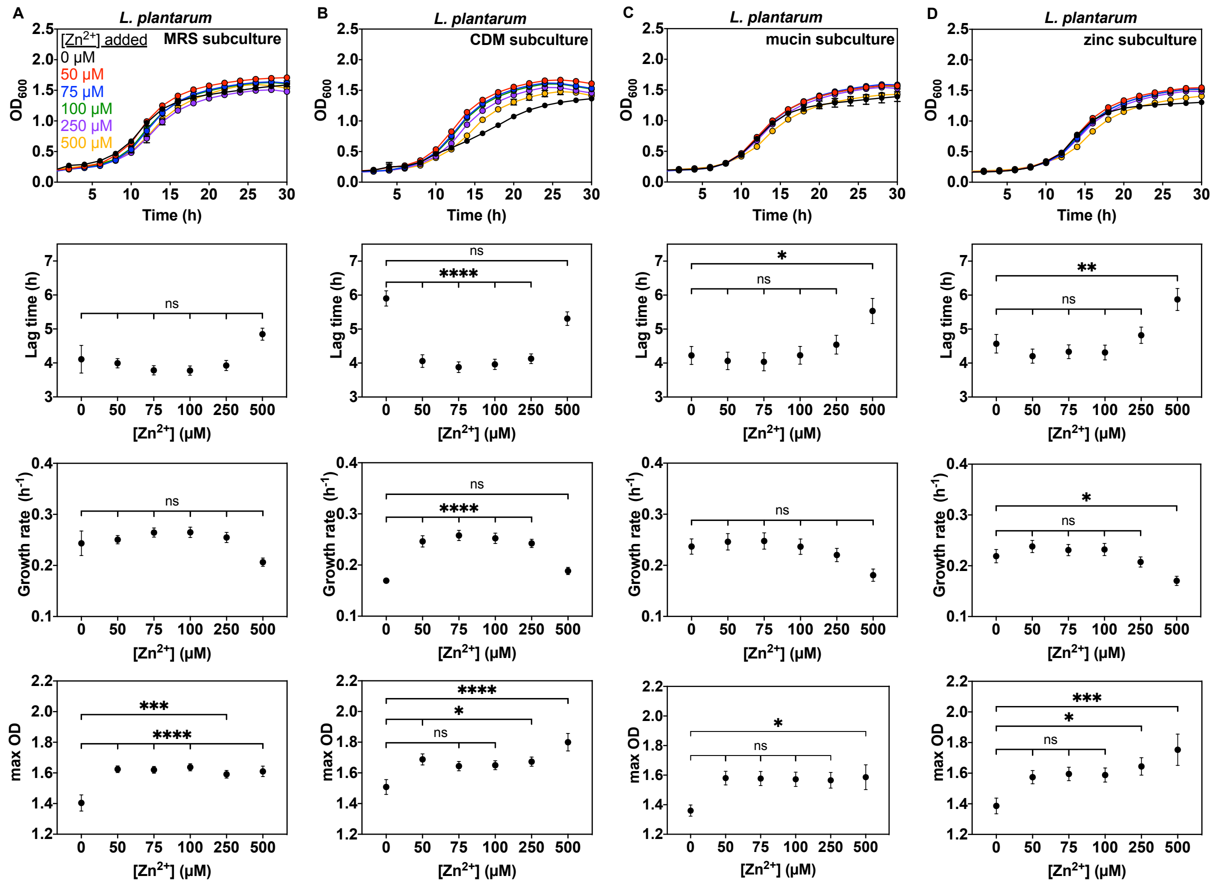

Figure S5. Effect of zinc on subculture growth for *L. plantarum* ATCC 14917 in CDM (Table S1) supplemented with 1% Wolfe trace mineral solution containing all metals except zinc (see Methods) and as measured by OD_{600}. MRS subculture was grown in MRS. CDM subculture was grown in CDM. Mucin subculture was grown with 0.1% mucin in CDM. Zinc subculture was grown with 100 μM $ZnSO_4$ in CDM (Scheme S1). Each of the above subcultures was then washed and grown in CDM (Table S1) supplemented with 1% Wolfe trace mineral solution containing all metals except zinc. Lag time, growth rate, and max OD beneath each representative growth curve represent the mean ± SEM of three biological replicates, each with ≥2 technical replicates. The growth parameters were calculated using nonlinear regression curve fitting to a 4-parameter Logistic equation with GraphPad Prism 9 software. ns (not significant), *p≤0.05, **p≤0.01, ***p≤0.001, ****p≤0.0001 as determined by one-way ANOVA with Tukey multiple comparison test.

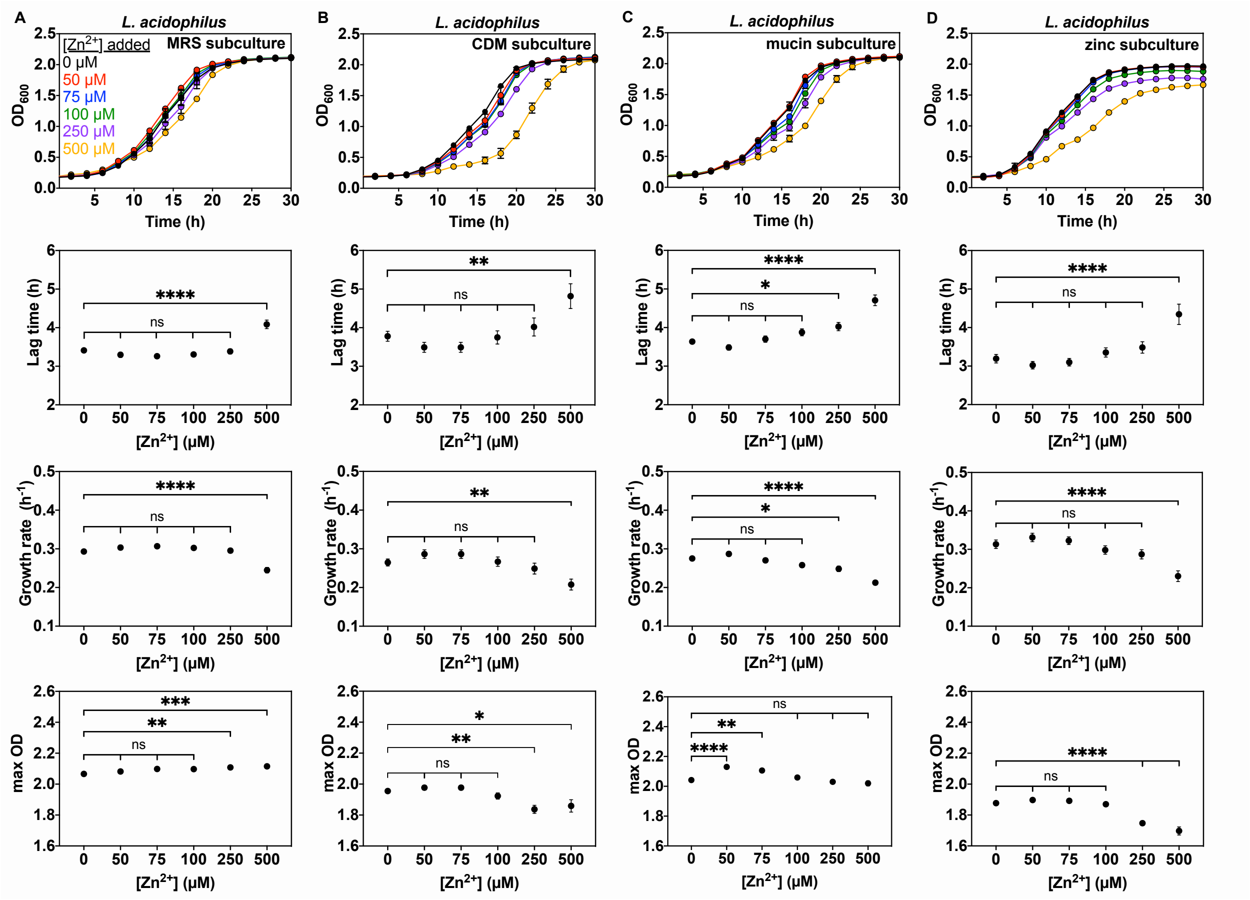

Figure S6. Effect of zinc on subculture growth for *L. acidophilus* ATCC 4356 in CDM (Table S1) supplemented with 1% Wolfe trace mineral solution containing all metals except zinc (see Methods) and as measured by OD_{600}. MRS subculture was grown in MRS. CDM subculture was grown in CDM. Mucin subculture was grown with 0.1% mucin in CDM. Zinc subculture was grown with 100 μM $ZnSO_4$ in CDM (Scheme S1). Each of the above subcultures was then washed and grown in CDM (Table S1) supplemented with 1% Wolfe trace mineral solution containing all metals except zinc. Lag time, growth rate, and max OD beneath each representative growth curve represent the mean ± SEM of three biological replicates, each with ≥2 technical replicates. The growth parameters were calculated using nonlinear regression curve fitting to a 4-parameter Logistic equation with GraphPad Prism 9 software. ns (not significant), *p≤0.05, **p≤0.01, ***p≤0.001, ****p≤0.0001 as determined by one-way ANOVA with Tukey multiple comparison test.

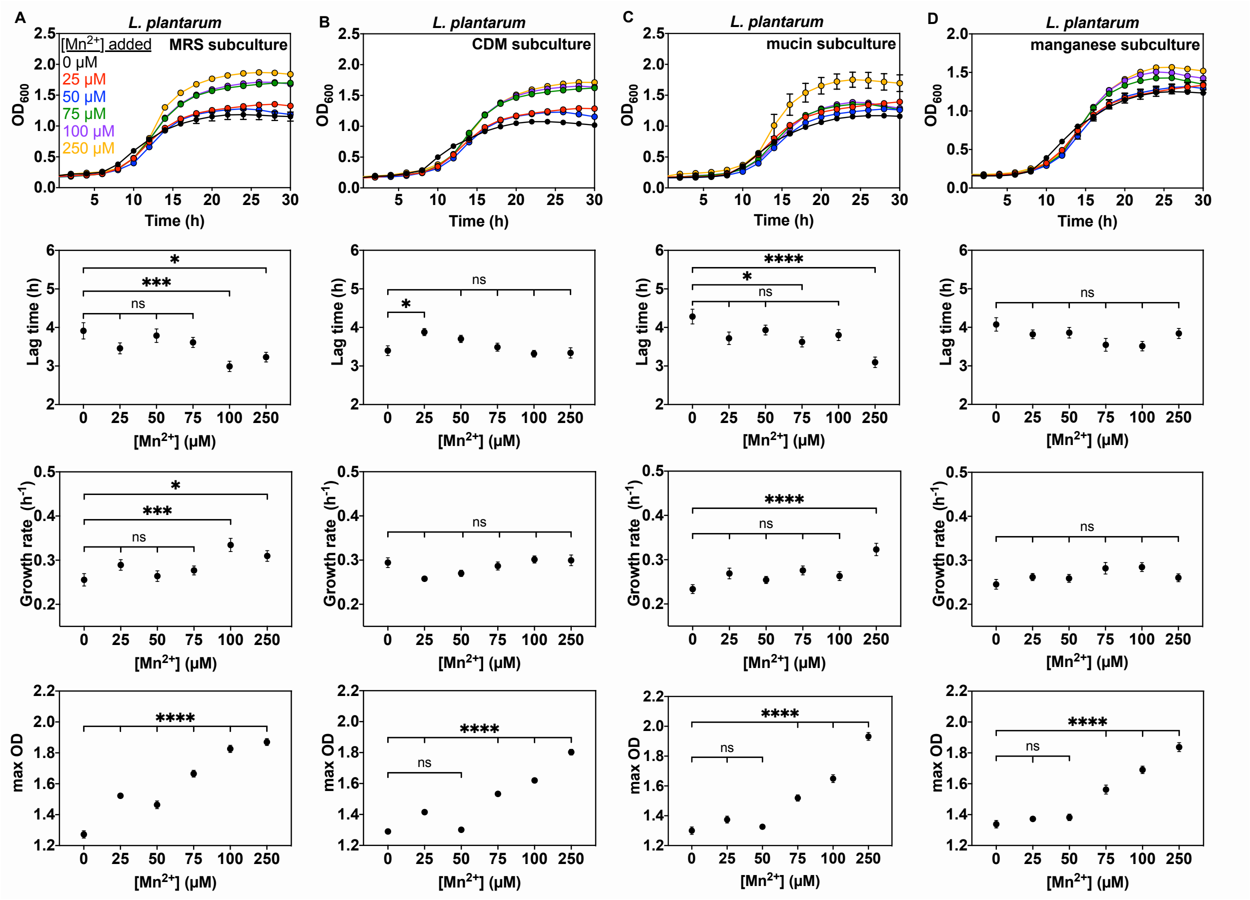

Figure S7. Effect of manganese on subculture growth for *L. plantarum* ATCC 14917 in CDM prepared without added manganese (Table S1) supplemented with 1% Wolfe trace mineral solution containing all metals except manganese (see Methods) and as measured by OD_{600}. MRS subculture was grown in MRS. CDM subculture was grown in CDM. Mucin subculture was grown with 0.1% mucin in CDM. Manganese subculture was grown with 50 μM $MnCl_2$ in CDM (Scheme S1). Each of the above subcultures was then washed and grown in CDM (Table S1) supplemented with 1% Wolfe trace mineral solution containing all metals except manganese. Lag time, growth rate, and max OD beneath each representative growth curve represent the mean ± SEM of three biological replicates, each with ≥2 technical replicates. The growth parameters were calculated using nonlinear regression curve fitting to a 4-parameter Logistic equation with GraphPad Prism 9 software. ns (not significant), *p≤0.05, **p≤0.01, ***p≤0.001, ****p≤0.0001 as determined by one-way ANOVA with Tukey multiple comparison test.

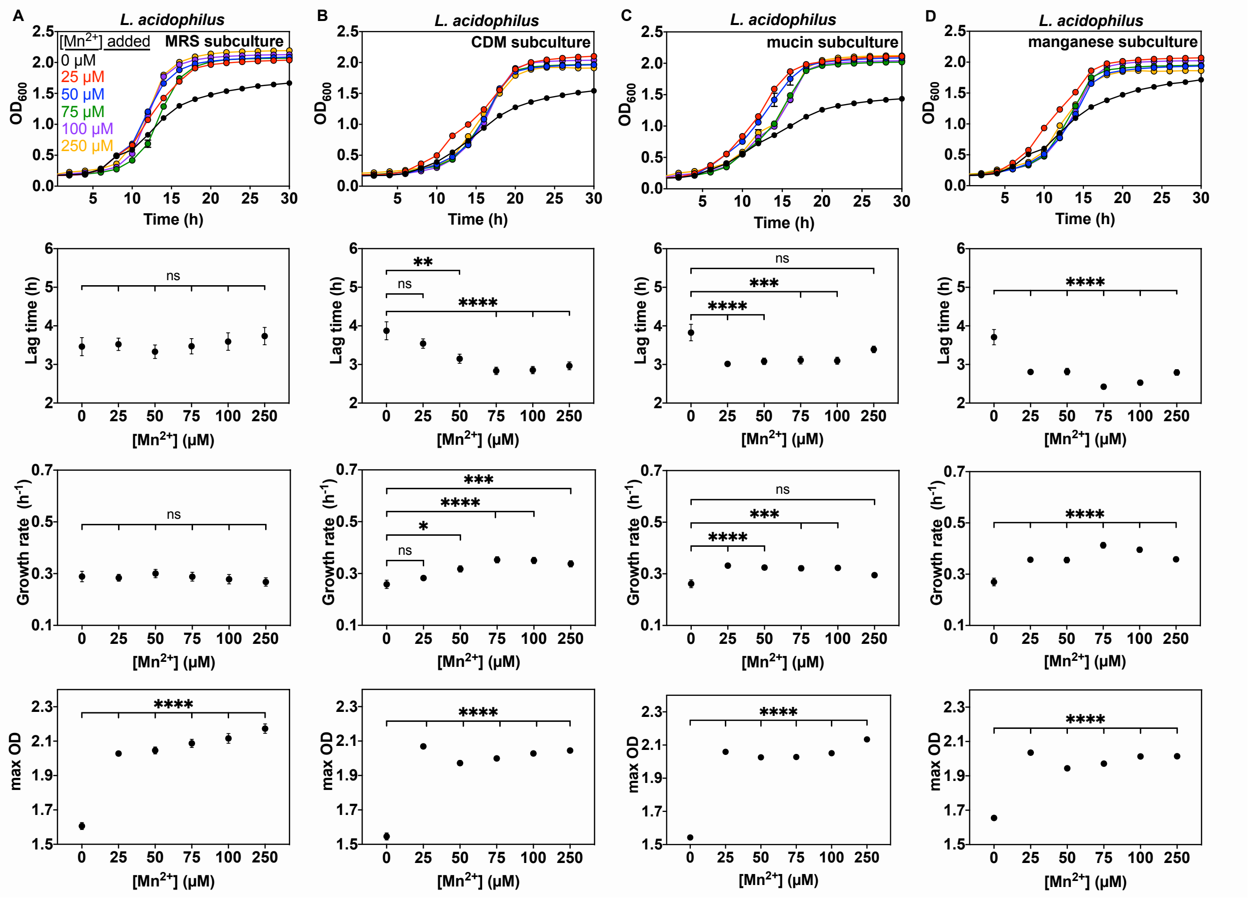

Figure S8. Effect of manganese on subculture growth for *L. acidophilus* ATCC 4356 in CDM prepared without added manganese (Table S1) supplemented with 1% Wolfe trace mineral solution containing all metals except manganese (see Methods) and as measured by OD_{600}. MRS subculture was grown in MRS. CDM subculture was grown in CDM. Mucin subculture was grown with 0.1% mucin in CDM. Manganese subculture was grown with 50 µM $MnCl_2$ in CDM (Scheme S1). Each of the above subcultures was then washed and grown in CDM (Table S1) supplemented with 1% Wolfe trace mineral solution containing all metals except manganese. Lag time, growth rate, and max OD beneath each representative growth curve represent the mean ± SEM of three biological replicates, each with ≥2 technical replicates. The growth parameters were calculated using nonlinear regression curve fitting to a 4-parameter Logistic equation with GraphPad Prism 9 software. ns (not significant), *$p \leq 0.05$, **$p \leq 0.01$, ***$p \leq 0.001$, ****$p \leq 0.0001$ as determined by one-way ANOVA with Tukey multiple comparison test.

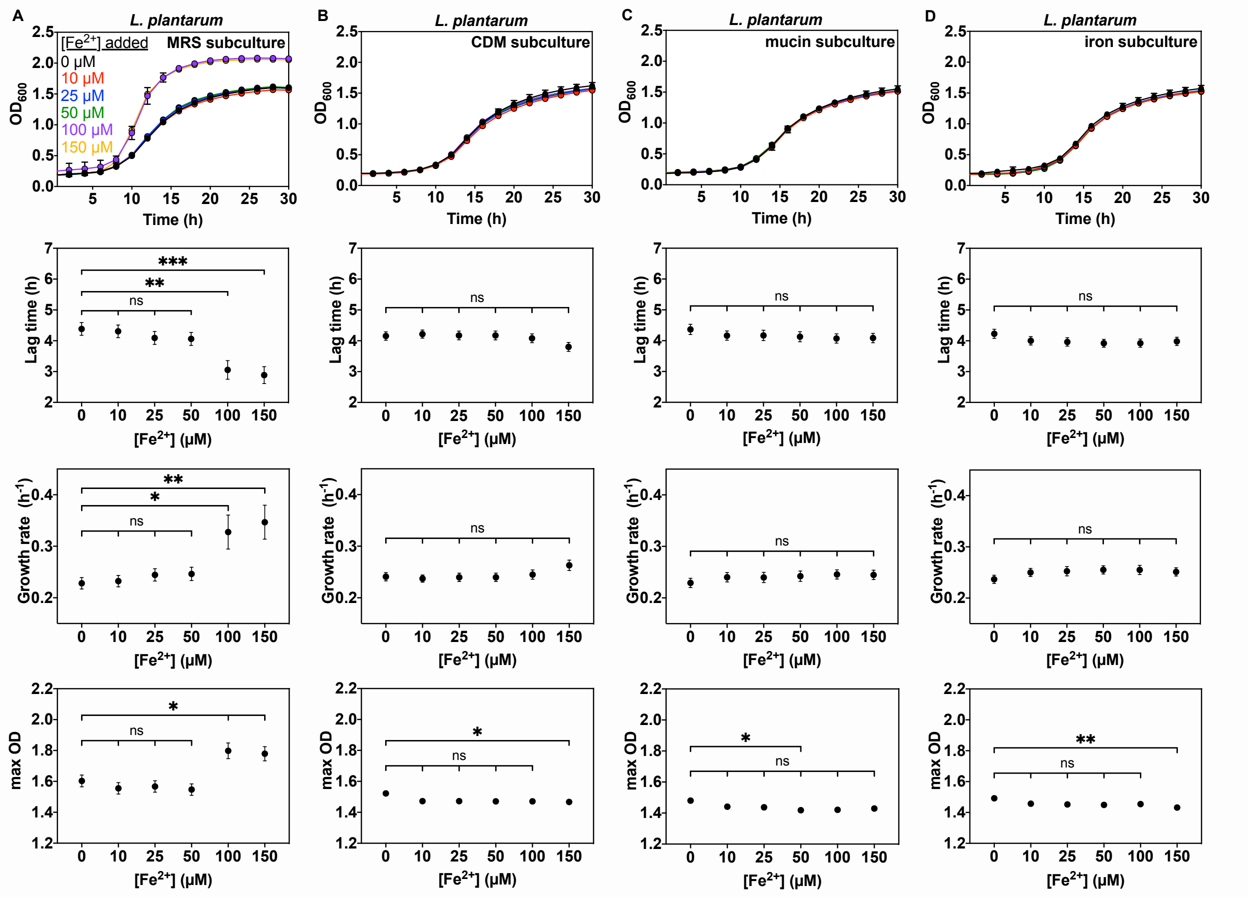

Figure S9. Effect of iron on subculture growth for *L. plantarum* ATCC 14917 in CDM (Table S1) supplemented with 1% Wolfe trace mineral solution containing all metals except iron (see Methods) and as measured by OD_{600}. MRS subculture was grown in MRS. CDM subculture was grown in CDM. Mucin subculture was grown with 0.1% mucin in CDM. Iron subculture was grown with 25 µM $FeSO_4$ in CDM (Scheme S1). Each of the above subcultures was then washed and grown in CDM (Table S1) supplemented with 1% Wolfe trace mineral solution containing all metals except iron. Lag time, growth rate, and max OD beneath each representative growth curve represent the mean ± SEM of three biological replicates, each with ≥2 technical replicates. The growth parameters were calculated using nonlinear regression curve fitting to a 4-parameter Logistic equation with GraphPad Prism 9 software. ns (not significant), *$p \leq 0.05$, **$p \leq 0.01$, ***$p \leq 0.001$ as determined by one-way ANOVA with Tukey multiple comparison test.

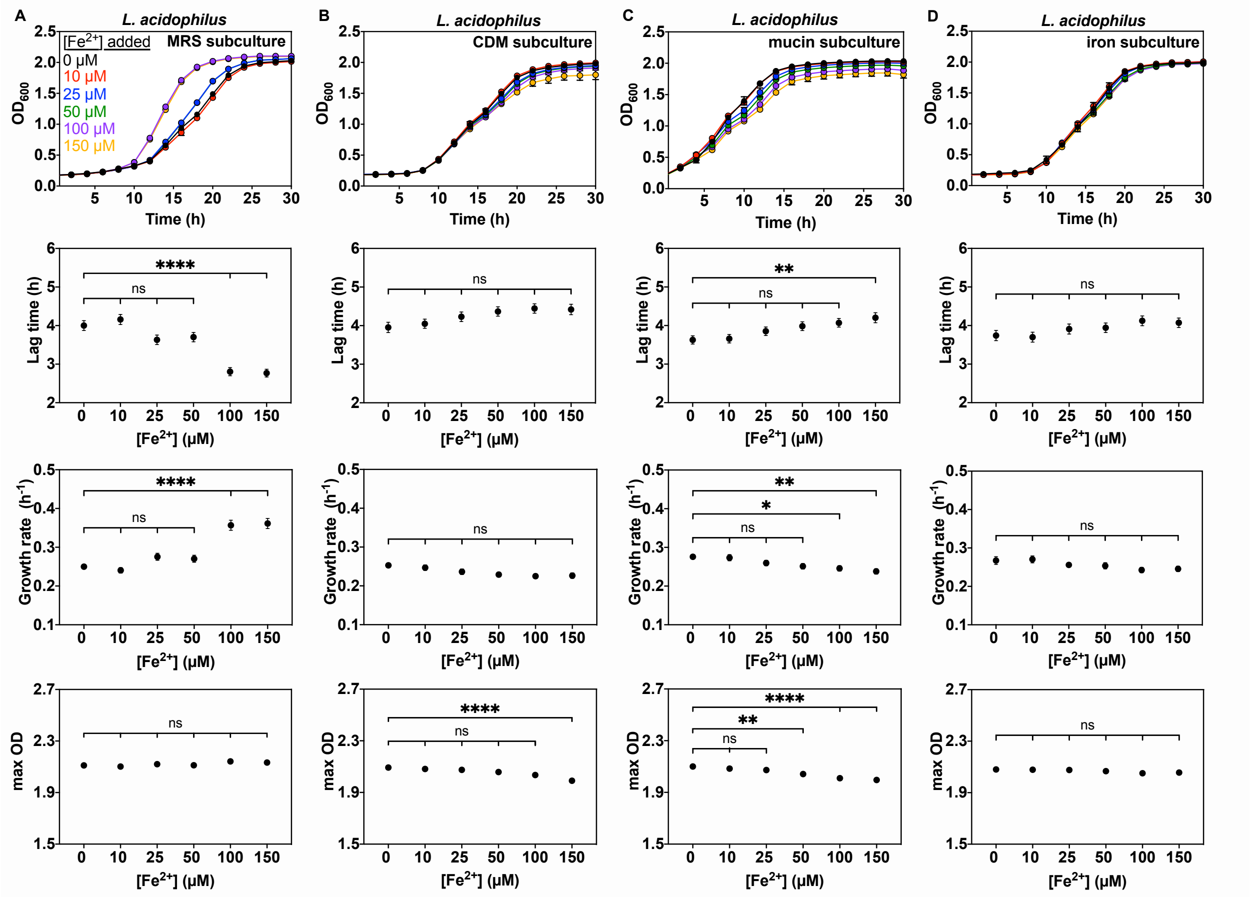

Figure S10. Effect of iron on subculture growth for *L. acidophilus* ATCC 4356 in CDM (Table S1) supplemented with 1% Wolfe trace mineral solution containing all metals except iron (see Methods) and as measured by OD_{600}. MRS subculture was grown in MRS. CDM subculture was grown in CDM. Mucin subculture was grown with 0.1% mucin in CDM. Iron subculture was grown with 25 μM $FeSO_4$ in CDM (Scheme S1). Each of the above subcultures was then washed and grown in CDM (Table S1) supplemented with 1% Wolfe trace mineral solution containing all metals except iron. Lag time, growth rate, and max OD beneath each representative growth curve represent the mean ± SEM of three biological replicates, each with ≥2 technical replicates. The growth parameters were calculated using nonlinear regression curve fitting to a 4-parameter Logistic equation with GraphPad Prism 9 software. ns (not significant), *$p \leq 0.05$, **$p \leq 0.01$, ****$p \leq 0.0001$ as determined by one-way ANOVA with Tukey multiple comparison test.

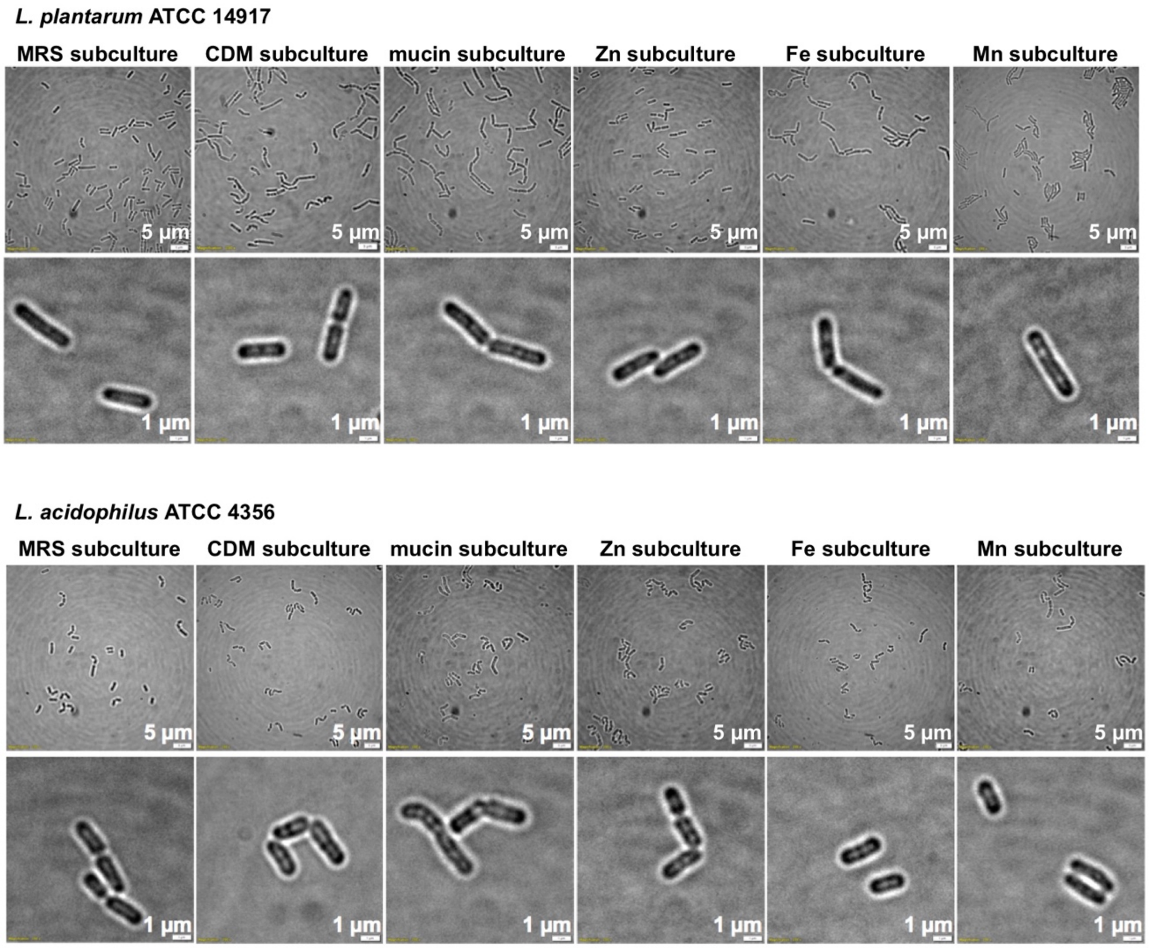

Figure S11. Bright-field microscopy images of lactobacilli grown in different subculture media. Mucin subculture was grown in 0.1% mucin in CDM. Zn subculture was grown in 100 µM $ZnSO_4$ in CDM. Fe subculture was grown in 25 µM $FeSO_4$ in CDM. Mn subculture was grown in 50 µM $MnCl_2$ in CDM.
